# A single cell transcriptomics map of paracrine networks in the intrinsic cardiac nervous system

**DOI:** 10.1016/j.isci.2021.102713

**Published:** 2021-07-19

**Authors:** Alison Moss, Shaina Robbins, Sirisha Achanta, Lakshmi Kuttippurathu, Scott Turick, Sean Nieves, Peter Hanna, Elizabeth H. Smith, Donald B. Hoover, Jin Chen, Zixi (Jack) Cheng, Jeffrey L. Ardell, Kalyanam Shivkumar, James S. Schwaber, Rajanikanth Vadigepalli

**Affiliations:** 1 Daniel Baugh Institute of Functional Genomics/Computational Biology, Department of Pathology, Anatomy, and Cell Biology, Thomas Jefferson University, Philadelphia, PA, USA; 2 University of California Los Angeles (UCLA) Cardiac Arrhythmia Center and Neurocardiology Research Program of Excellence, Department of Medicine, UCLA, Los Angeles, CA, USA; 3 Department of Biomedical Sciences, James H. Quillen College of Medicine, East Tennessee State University, Johnson City, TN, USA; 4 Burnett School of Biomedical Sciences, College of Medicine, University of Central Florida, Orlando, FL, USA

**Keywords:** Cardiovascular medicine, Molecular physiology, Systems neuroscience, Transcriptomics

## Abstract

We developed a spatially-tracked single neuron transcriptomics map of an intrinsic cardiac ganglion, the right atrial ganglionic plexus (RAGP) that is a critical mediator of sinoatrial node (SAN) activity. This 3D representation of RAGP used neuronal tracing to extensively map the spatial distribution of the subset of neurons that project to the SAN. RNA-seq of laser capture microdissected neurons revealed a distinct composition of RAGP neurons compared to the central nervous system and a surprising finding that cholinergic and catecholaminergic markers are coexpressed, suggesting multipotential phenotypes that can drive neuroplasticity within RAGP. High-throughput qPCR of hundreds of laser capture microdissected single neurons confirmed these findings and revealed a high dimensionality of neuromodulatory factors that contribute to dynamic control of the heart. Neuropeptide-receptor coexpression analysis revealed a combinatorial paracrine neuromodulatory network within RAGP informing follow-on studies on the vagal control of RAGP to regulate cardiac function in health and disease.

## Highlights

• Spatially-tracked single neuron transcriptomics map of intrinsic cardiac ganglia
• Distinct composition of intrinsic cardiac neurons compared to the central neurons
• Correlation of cholinergic and catecholaminergic markers suggesting neuroplasticity
• Adaptive and dynamic system driven by putative paracrine neuromodulatory networks

## Graphical abstract

**Figure:**
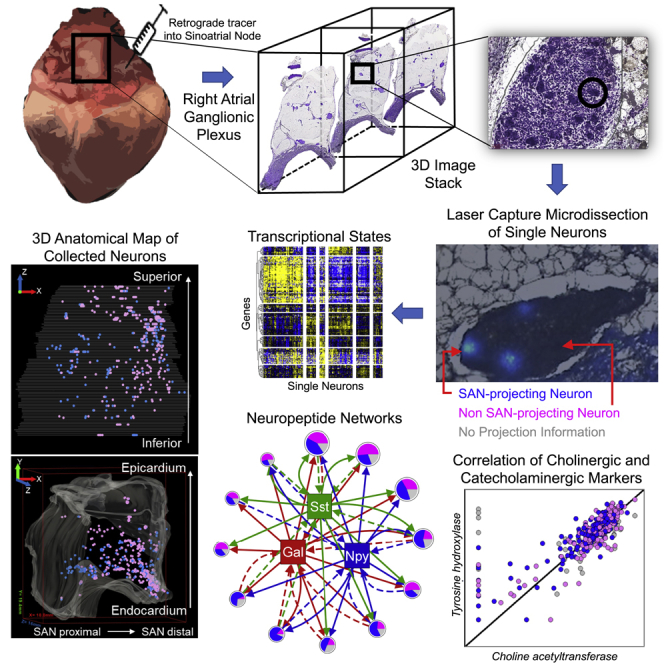

## Introduction

In this study, we performed a spatially-tracked single-cell transcriptomic analysis of an intrinsic cardiac ganglion in the pig heart to uncover the complex molecular landscape and putative paracrine neuromodulatory networks. The functional significance and complexity of the intrinsic cardiac nervous system (ICNS) has been studied for years with the majority of the focus on the physiological aspects. The ganglia at the heart are thought to constitute a “little brain” with afferent, parasympathetic and sympathetic components (Armour, 2008; Hoard et al., 2007; Neel and Parsons, 1986; Parsons et al., 1987; Wake and Brack, 2016; Weihe et al., 2005). Physiological studies using epicardial ablation have demonstrated that the intrinsic cardiac ganglia mediate central control of cardiac function through vagal and sympathetic circuits (Berger et al., 2019; Driessen et al., 2016; Qin et al., 2017). Yet, little is known about the distribution and organization of the molecular profiles of the neurons constituting the intrinsic cardiac ganglia. Recent single neuron gene expression profiling studies have uncovered the wide range of molecularly-defined subtypes (Hu et al., 2017; Kupari et al., 2019), gradient-based organization driven by inputs to neurons (Park et al, 2014, 2016; Shen et al., 2012), as well as molecular plasticity during homeostatic conditions and physiological perturbations (Dulcis et al., 2013; Park et al, 2014, 2016) in the central nervous system (CNS) as well as in the peripheral ganglia (Kupari et al., 2019). We set out to pursue such approaches and combine them with 3D positional information within the tissue to develop an extensive spatially-tracked molecular map of the ICNS.

In the present study, we focus on the RAGP as a key control point in the circuit that mediates vagal modulation of the SA node (SAN) activity. The routes of parasympathetic and sympathetic control of SAN were determined by several studies; surgical ablation of different areas of the heart and vagal stimulation showing a shift in pacemaker activity (Bouman et al., 1968; Geis et al., 1973; Randall and Ardell, 1985). Stimulation of the RAGP fat pad was shown to cause a reduction in heart rate in dogs and human patients and cause a pacemaker shift (Butler et al., 1990; Carlson et al., 1992; Furukawa et al., 1990). Later studies ablating RAGP showed that this group of neurons is critical to the cardiac pacemaker response to vagal stimulation (McGuirt et al., 1997). Molecular analysis of RAGP, and ICNS in general, has largely been targeted at the protein level using immunolabeling. The majority of the cardiac ganglia have been found to be cholinergic (ChAT+) (Hoard et al, 2007, 2008; Horackova et al., 2000; Richardson et al., 2003; Rysevaite et al., 2011), while the proportion of observed catecholaminergic (TH+) neurons was widely variable across studies (Hoard et al., 2008; Hoover et al., 2009; Richardson et al., 2003; Rysevaite et al., 2011). Additionally, some studies have reported co-expression of ChAT and TH in 10–20% of ICNS neurons (Rysevaite et al., 2011; Zarzoso et al., 2013). The expression of other neurotransmitter/neuromodulator systems such as NPY, GAL, and SST have also been described within ICNS (Day et al., 1985; Habecker et al., 2005; Herring, 2015; Herring et al., 2012; Hökfelt et al., 1977; Moriarty et al., 1992). Here, we undertake a broad-based survey to characterize the gene expression of a wide range of neuromodulators underlying the dynamic neuronal control of cardiac function and their co-expression in single neurons within the RAGP.

In a recent proof-of-principle study, we demonstrated a coordinated experimental approach that integrates imaging technologies with high throughput gene expression data (HT-qRTPCR) to develop a 3D anatomical and molecular map of rat ICNS (Achanta et al., 2020). Here, we build on that approach to incorporate single-cell scale RNA-seq and precisely integrate molecular data into a digitally reconstructed 3D RAGP with anatomical context of the pig heart. This approach contrasts with that of typical droplet-based single-cell transcriptomics techniques in that the spatial and anatomical information of each sample is extensively tracked, which allows mapping of the molecular information into a 3D-reconstructed anatomical organization of the tissue. Such an integrated anatomical-molecular map permits analysis of relationships between spatial location and molecular profiles, within the tissue as well as reference to adjacent anatomical features (Achanta et al., 2020; Park et al., 2016). We now find consistent neuroanatomical structure and diverse molecular properties of ganglia in the ICNS of a large porcine mammal, accepted as close to human. Our newly developed data demonstrate that the local cardiac ganglia harbor anatomical and molecular features necessary to function as complex signal processing units that critically mediate vagal control of heart function and health (Hanna et al., 2021).

## Results

### Mapping spatially-tracked single-cell transcriptomics onto an imaging-based 3D tissue reconstruction of pig right atrial ganglionic plexus

**Figure 1:**
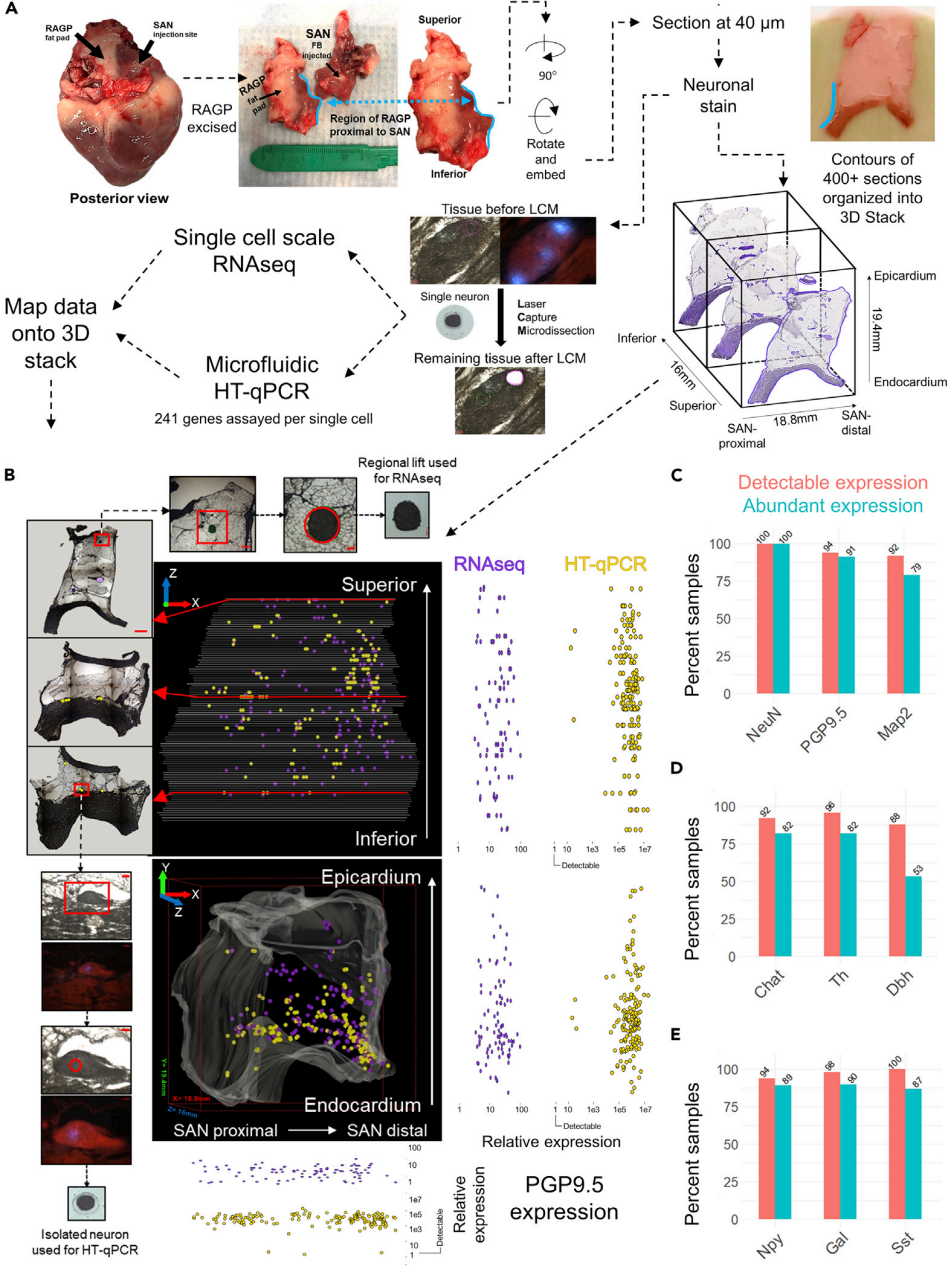
Mapping spatially-tracked single-cell transcriptomics onto an imaging-based 3D tissue reconstruction of pig right atrial ganglionic plexus (RAGP) (A) Integrated workflow starting from injection of neuronal tracer into the sinoatrial node (SAN) region of the pig heart, followed by isolation, embedding, and cryosectioning of the RAGP, acquisition of block face images for 3D reconstruction, staining for neuronal localization within the tissue, and obtaining spatially-tracked single neuron samples via laser capture microdissection (LCM) for downstream processing using RNA-seq and high-throughput real-time PCR (HT-qPCR), yielding transcriptomic data that is mapped onto a 3D anatomical framework. Scale bars: 50 μm. (B) Representative visualization of the 3D anatomical framework of an RAGP depicting the location of spatially-tracked single neurons sampled via LCM (purple dots - neuronal samples for RNA-seq; yellow dots - neuronal samples for HT-qPCR). The cross-sections of the stack show the corresponding tissue sections from which the neuronal samples were obtained. Scale bars: whole tissue sections, 500 μm; regional lift zoom 1, 500 μm; regional lift zoom 2, 100 μm; isolated neuron zoom 1, 100 μm; isolated neuron zoom 2, 50 μm. The relative expression of *PGP9.5* in these spatially-tracked neuronal samples is shown with reference to the axes of the 3D stack. The bounding box on the lower panel shows 18.8 mm, 19.4 mm, and 16 mm on the x, y, and z axis, respectively. (C–E) Proportion of samples that showed detectable and abundant expression of select pan-neuronal markers (C), cholinergic and catecholaminergic markers (D), and neuropeptides (E), as assessed by HT-qPCR. Data shown is based on combining 405 single neuron samples across n = 4 animals.

We developed a 3D map of the single neuron scale gene expression within the pig RAGP. We used our recently developed method pipeline that combines single neuron anatomical position using 3D mapping with gene expression data of the mapped neurons obtained from single-cell scale RNA-seq and high-throughput qRT-PCR (HT-qPCR) (Achanta et al., 2020) (Figure 1A). The RAGP neurons that project to SAN were labeled by a tracer that was injected into the SAN 3 weeks prior to sacrificing the animal for tissue harvesting. The heart tissue corresponding to the location of RAGP was sectioned serially from superior to inferior end for laser capture microdissection (LCM) of single neurons and neuron pools (n = 4 animals). During sectioning, blockface images were obtained, which were contoured and organized into a 3D image stack (2,698 images across n = 4 animals). After sectioning and staining, the tissue was subjected to LCM where both FastBlue labeled (SAN-projecting) and unlabeled (considered as Non SAN-projecting in the present analysis) single neurons were collected for microfluidic HT-qPCR (405 single neurons X 241 genes per neuron across 4 RAGP) and regional neuronal lifts were collected for single-cell scale RNA-seq (90 neuron pools from one RAGP). Image tracking was used to digitally annotate the spatial locations for single-cell neuronal samples on a digitally reconstructed RAGP using TissueMapper software (Figure 1B, Video S1). Expression levels of *Uchl1* (*PGP9.5)*, a pan neuronal marker, showed a wide range that is persistent throughout the RAGP with no spatial bias for enhancement or depletion (Figures 1B and 1C). We assessed all single neuron samples for expression of typical neuronal markers, cholinergic and catecholaminergic markers, and key neuropeptides (Figures 1C–1E). Nearly 100% of all collected samples not only showed detectable expression but also abundant expression of *NeuN*, a common neuronal marker (Gusel'nikova and Korzhevskiy, 2015). *PGP9.5* and *Map2,* another neuron-specific gene (Schofield et al., 1995; Soltani et al., 2005), were also abundantly expressed in a large majority of the sampled single neurons (Figure 1C). A high percentage of neurons showed abundant expression of *Chat*, *Th*, and to some extent, *Dbh*, suggesting a high degree of co-expression between cholinergic and catecholaminergic markers across single RAGP neurons (Figure 1D). Neuropeptides such as Neuropeptide Y (*Npy*), Galanin (*Gal*), and Somatostatin (*Sst*) also showed abundant expression in a high proportion of neurons in the RAGP (Figure 1E).

Video S1. Acquisition of single neurons from the RAGP and 3D visualization of SAN-projecting and non SAN-projecting neurons within a representative RAGP, related to Figure 3

### Transcriptomic landscape of pig RAGP from a single-cell scale RNAseq analysis

**Figure 2:**
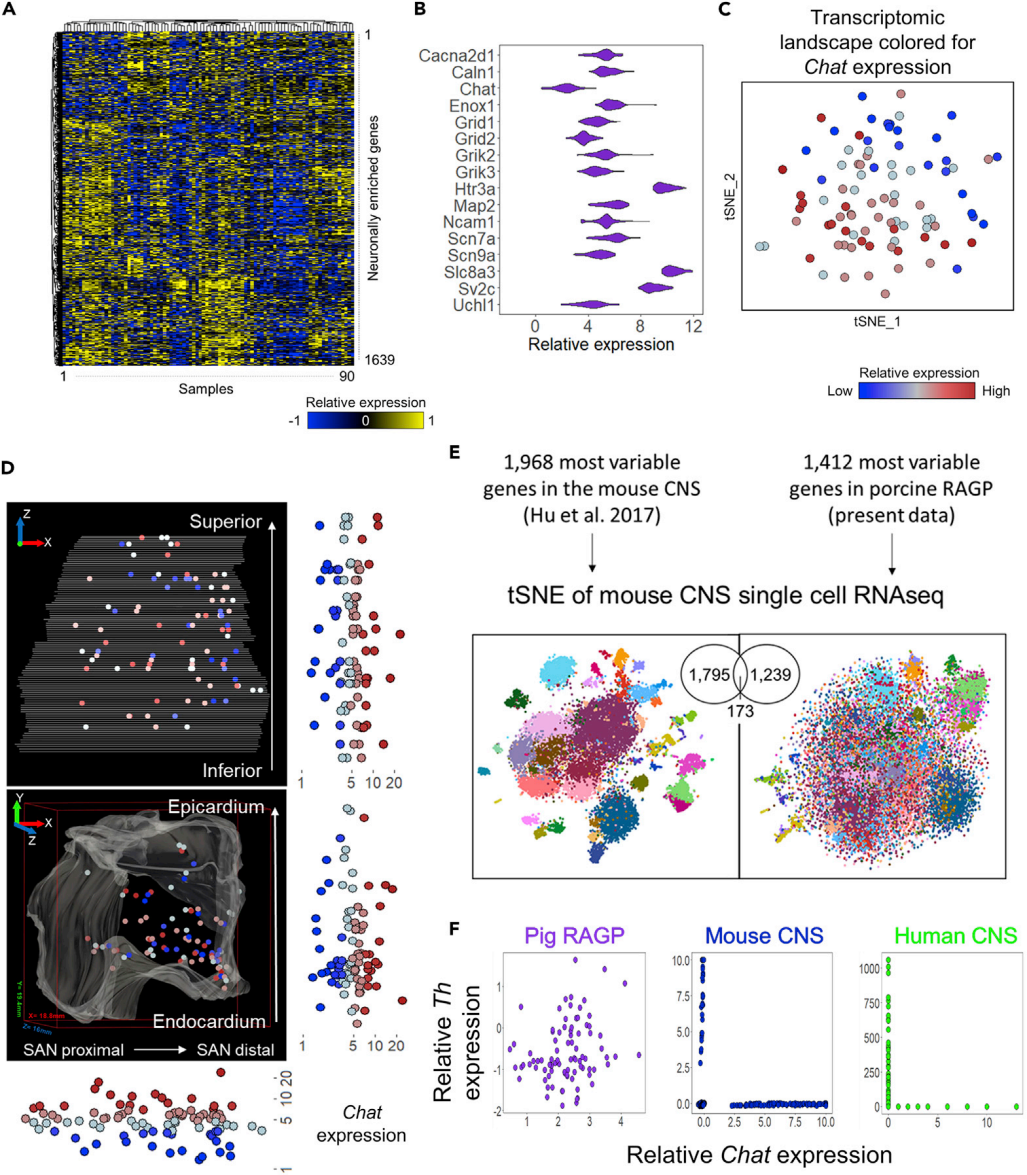
Transcriptomic landscape of pig RAGP from a single-cell scale RNAseq analysis (A) Expression of 1,882 neuronally-enriched genes in 90 spatially-tracked neuronal clusters in RAGP based on single-cell scale RNA-seq profiling of laser capture microdissected samples. The genes included in the heatmap were selected by analyzing the GTEx database for those enriched in the neuronal tissues compared to other tissue types. Of the genes identified as neuronally enriched in the GTEx database, 1,639 genes were present in the RAGP neurons. (B) Distribution of select abundantly expressed neuronal genes. (C) Transcriptomic landscape as delineated by tSNE indicating the gradient of *Chat* throughout a distributed cloud. (D) Visualization within the 3D anatomical framework for a representative RAGP. The relative expression of choline acetyltransferase (*Chat*) in these spatially-tracked neuronal samples is shown with reference to the axes of the 3D stack. The bounding box on the lower panel shows 18.8 mm, 19.4 mm, and 16 mm on the x, y, and z axis, respectively. (E) A comparison of the distribution of CNS neuronal types based on the most-variable genes in mouse CNS (Hu et al., 2017) versus in pig RAGP (present data). The tSNE plots are colored based on 40 distinguishable mouse CNS neuronal states described in Hu et al. (2017). (F) Scatterplots comparing the expression of *Th* vs *Chat* in the pig RAGP (present data), mouse CNS (Hu et al., 2017), and human CNS (Human Multiple Cortical Areas SMART-seq, 2019) (https://portal.brain-map.org/atlases-and-data/rnaseq/human-multiple-cortical-areas-smart-seq).

142 regional neuronal samples from the RAGP of a female Yucatan minipig were collected through LCM and spatially tracked as described above and subjected to single-cell scale RNA-seq using the Smart-3SEQ protocol (Foley et al., 2019) (described in STAR methods). After quality check we were left with 90 samples with detectable expression in 15,000 genes. Using publicly available data in the GTEx database (GTEx Consortium, 2013), we retrieved a list of genes that are statistically enriched in neuronal tissues compared to other tissue types (p< 0.01). A total of 1,639 neuronally enriched genes showed detectable expression in our RNA-seq data (Figure 2A). Of note, genes for ion channels associated with calcium signaling as well as glutamatergic receptors were expressed at high levels throughout the RAGP (Figure 2B).

The transcriptomic landscape of the neuronally enriched sample set was assessed through a nonlinear embedding approach, t-stochastic neighbor embedding (tSNE) (Maaten and Hinton, 2008). The results show that the samples were not separated into groups corresponding to distinguishable phenotypes, but were instead organized as a single cloud suggesting a gradient of gene expression based organization of underlying neuronal molecular states (Park et al., 2014). Coloring the tSNE map for relative expression of *Chat,* an important marker for cholinergic expression, reveals a distinct gradient across the transcriptomic landscape (Figure 2C). We then visualized the expression pattern of *Chat* in the context of 3D anatomical space. Similar to the spatially distributed expression of *PGP9.5* (Figure 1B), *Chat* expression was widely distributed throughout the RAGP with no spatial gradients along any axis (Figure 2D).

We compared the transcriptomic profiles of the RAGP neurons with the molecular phenotypes identified from single neuron transcriptomics analysis in the CNS (Hu et al., 2017). We used the available mouse CNS data, due to lack of comparable pig CNS single neuron transcriptomic dataset. We reconstructed the tSNE map of single neurons in the mouse CNS based on the 1,968 expression of the highly variable genes (Hu et al., 2017) (Figure 2E). To compare how the neuronal phenotypes identified in the CNS compared to those in the RAGP, we first extracted genes contributing most to the variability in the RAGP through principal component analysis (PCA). Of the 1,814 most variable genes in the RAGP, a subset of 1,412 genes were found in the mouse CNS single neuron data (Hu et al., 2017). Visualization by tSNE map shows that the well-defined neuronal clusters based on variable genes in the CNS are largely lost when the neuronal heterogeneity was analyzed using the 1,412 most variable genes in the RAGP (Figure 2E). These results demonstrate that the specific gene markers that delineate the variability in the RAGP are not similarly variable in the CNS, and vice versa, suggesting a different organization of neuronal heterogeneity between RAGP and CNS structures.

Considering that a large proportion of neurons showed expressions of *Th* and *Chat* (Figure 1D), we examined the co-expression patterns of these genes in the transcriptomics data. Neurons in the mouse (Hu et al., 2017) and human CNS (Human Multiple Cortical Areas SMART-seq, 2019) show mutually exclusive expression of *Th* and *Chat*. In stark contrast to the lack of *Th* and *Chat* co-expression in the CNS, we found a high degree of co-expression between *Th* and *Chat* in the RAGP neurons (Figure 2F). This finding further augments the results seen in the tSNE map (Figure 2E) suggesting that the neuronal molecular states within the RAGP may be organized in a manner that is not similar to the neuronal phenotypes observed in the CNS. We further compared these co-expression patterns against other available data in the peripheral nervous system (PNS). Comparing expression of *Th* and *Chat* in different branches of the mouse PNS (Zeisel et al., 2018), as well as in different cell phenotypes in sympathetic ganglia (Furlan et al., 2016), revealed patterns of mutual exclusivity similar to those observed in the human and mouse CNS (Figure S1). These findings further highlight the unique molecular architecture of the RAGP.

### Landscape of neuronal transcriptional states in the pig RAGP

**Figure 3:**
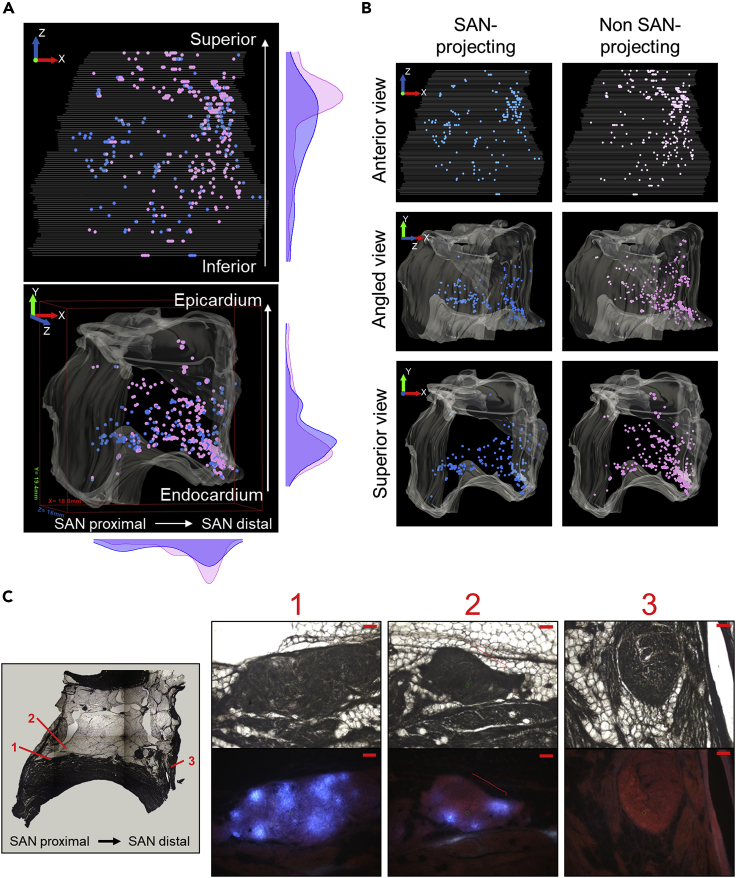
Broad RAGP anatomy of SAN-projecting and non SAN-projecting neurons (A) Visualization within the 3D anatomical framework of both SAN-projecting (blue) and non SAN-projecting (purple) neurons that were comprehensively identified in select sections of a representative RAGP. Panels along the right side and bottom show density plots representing the density of projecting and non-projecting neurons along each axis. The bounding box on the lower panel shows 18.8 mm, 19.4 mm, and 16 mm on the x, y, and z axis, respectively. (B) Anterior (top), angled (middle) and superior (bottom) views of a representative RAGP showing only the SAN-projecting neurons (left) or non SAN-projecting neurons (right). The X, Y, Z measurements are consistent with those in panel (A). (C) A select section of the RAGP (7,040 μm from the superior aspect) zooming in on three different neuron clusters showing a high percentage of neurons within the cluster projecting to the SAN toward the SAN-proximal side of the RAGP (1), a cluster with no SAN-projecting neurons toward the SAN-distal side of the RAGP (3) and a cluster with a mix of both projecting and non-projecting neurons in between (2). Scale bars: 100 μm. Tissue measured 18.8 mm from left to right (xaxis) and 19.4 mm top to bottom (yaxis). For an animated visualization see Video S1.

Using the 3D map of a representative RAGP, examination at the single-cell scale allowed us to visualize the distribution of neurons within their 3D anatomical framework, revealing that neurons, while distributed throughout the RAGP, are more densely packed closer to the endocardium (Figure 3A). Annotation of neurons based on their projection to the SAN indicates that while both projecting and non-projecting neurons are present throughout the RAGP, SAN-projecting neurons appear to be more concentrated closer to the SAN and less concentrated toward the epicardium (Figures 3A–3C, Video S1). We assayed 405 spatially-tracked single neuron samples from RAGP (n = 4 animals) for expression of 211 genes within each neuron using HT-qPCR, representing both SAN-projecting and non SAN-projecting neurons (Figure S2A). At the molecular level, a set of only 6 genes (*Cck, Gal, Grp, Hcrtr1, Ntrk1, Ret*) showed significant differences in the distribution of expression between SAN-projecting and non SAN-projecting neurons (K-S statistic, FDR-adjusted p< 0.01, fold change >2, Figure S2B). Interestingly, expression of *Cck*, *Gal*, and *Hcrtr1* have recently been examined within the peripheral nervous system where expression of *Hcrtr1* and *Gal* has been shown in sympathetic neurons in the PNS while neuropeptides *Cck* and *Gal* have been shown in enteric neurons (Zeisel et al., 2018). Meanwhile, *Ret* and *Ntrk1* have proven crucial for differentiation of neuronal subtypes in sympathetic ganglia (Furlan et al., 2016), although the significance of their differential expression between SAN-projecting and non SAN-projecting neurons is still unclear. Comparing neurons across all 4 RAGP, the expression distribution of select neuronal markers *NeuN, PGP9.5, Chat, Th, Dbh,* and *Npy* were relatively consistent across animals and between SAN-projecting and non SAN-projecting neurons (Figure S2C).

**Figure 4:**
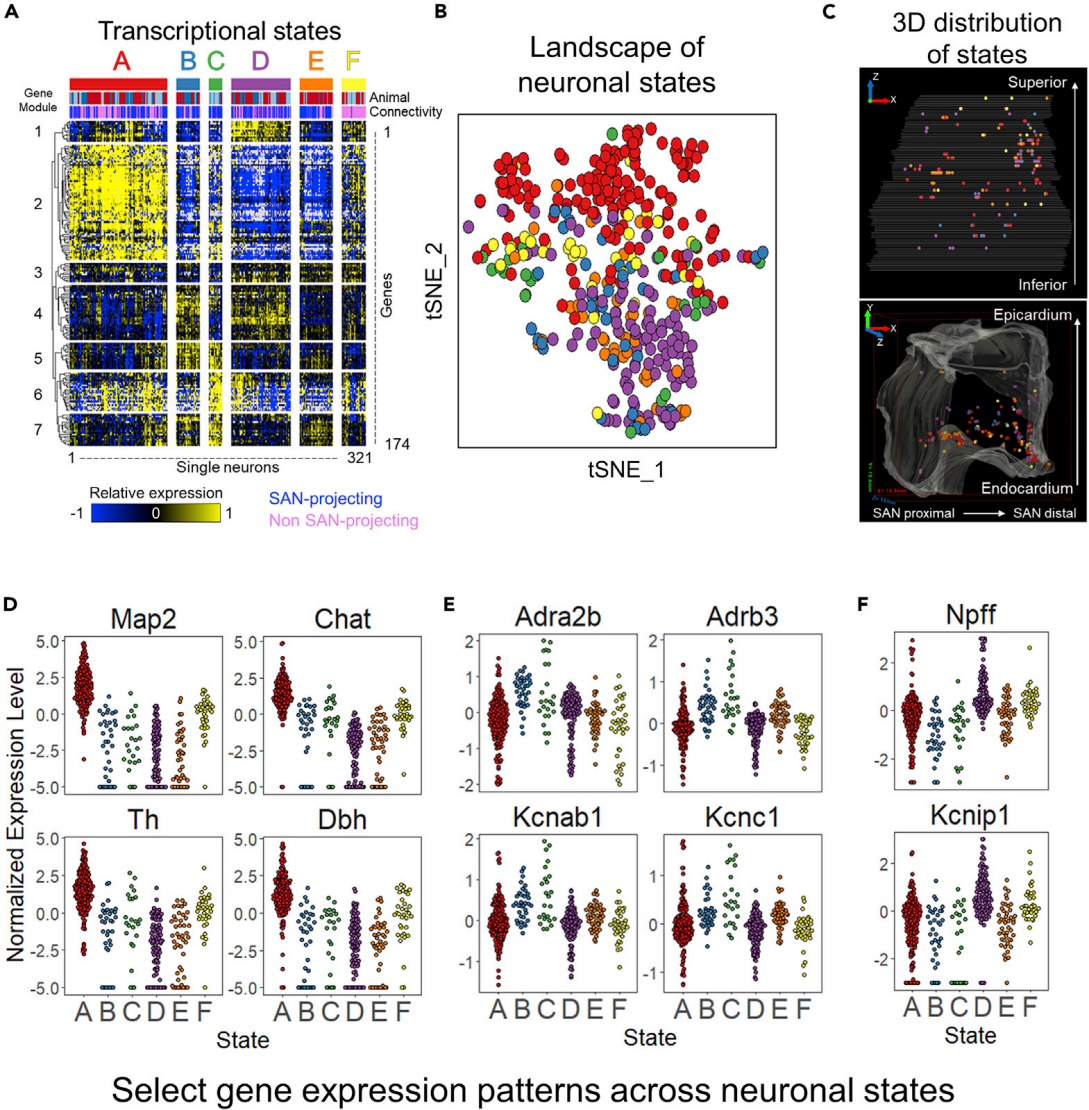
Landscape of neuronal transcriptional states in the pig RAGP (A) Expression of 174 genes, each assayed in 321 single neurons (n = 3 animals) through HT-qPCR, yielding six transcriptional states using a combination of clustering and template matching analysis. A majority of the states consisted of both SAN-projecting and non SAN-projecting neurons. Sample annotations at the top of the heatmap indicate whether the single neurons were SAN-projecting or non SAN-projecting and indicate the distribution across animals. A complete heatmap with 405 neurons from all 4 RAGP is shown in Figure S3. (B) Landscape of neuronal transcriptional states visualized as a tSNE plot. Colors correspond to the states shown in panel (A). (C) Visualization of the neuronal states within the 3D anatomical framework for a representative RAGP. The bounding box on the lower panel shows 18.8 mm, 19.4 mm, and 16 mm on the x, y, and z axis, respectively. (D–F) Expression distribution of select genes with enrichment in specific transcriptional states: (D) state (A)*Map2*, *Chat*, *Th*, *Dbh*; (E) states B and C*Adrab2b*, *Adrb3*, *Kcnab1*, *Kcnc1*; and (F) states D and F*Npff* and *Kcnip1*. Enrichment assessed by a one-way ANOVA and post hoc Tukey Honest Significant Difference test pvalue < 0.01.

In order to identify gene expression modules that can better characterize the neurons based on the connectivity to SAN, we used a combination of clustering and template matching. This robust approach mimics algorithms such as k-means that seed certain clusters and map the data into the seeds taken from an unbiased hierarchical clustering approach, yielding six transcriptional states within the RAGP neurons (Figure 4A). Briefly, the gene expression profiles of SAN-projecting neurons from one male and one female RAGP were subjected to hierarchical clustering to partition the single neurons into distinct states. These states were used as templates to assign the remaining non SAN-projecting neurons to one of these states based on correlation to the template, with neurons below the correlation threshold sequestered into an additional state. Neurons from a different pair of male and female RAGP were sorted into these transcriptional states based on correlation to the template profile, revealing similar patterns (Figures 4A andS3). Examination of these transcriptional states reveals that the molecular signatures of SAN-projecting and non SAN-projecting neurons are remarkably similar. A closer examination uncovered a state consisting of almost entirely SAN-projecting neurons (state C), and another state consisting of almost entirely non SAN-projecting RAGP neurons (state F). Notably, neither of these states could be distinguished from the other neuronal states by the patterns of any given gene expression module. Instead, each state was characterized by a combinatorial pattern of multiple gene expression modules (Figure 4A). Visualization using a tSNE map revealed that within the context of the transcriptional landscape, these neuronal states are distributed across parts of a single cloud, suggesting a gradient-based organization of the neuronal states (Figure 4B). Analysis of the potential patterns and gradients of these states within the 3D anatomical framework of a representative RAGP revealed that the neuronal states were evenly distributed throughout the RAGP (Figure 4C, Video S2).

Video S2. Visualization of identified transcriptional states within the 3D anatomical framework of a representative RAGP, related to Figure 4

We further explored the heterogeneity of gene expression distribution across these neuronal states (Figures 4D–4F, Figure S4A). State A is largely defined by high expression of genes in module 2 which include neuron-specific genes such as *Map2, Eno2,* and *PGP9.5* as well as genes involved in neurotransmitter processes such as *Chat*, *Th*, and *Dbh* (Figure 4D). States B and C were both characterized by high expression of genes in modules 5 and 7, which include several adrenergic receptors and potassium channels (Figure 4E). Several adrenergic receptors were expressed abundantly within RAGP neurons (Figure S5A). Interestingly, the beta-adrenergic receptors, which are of particular importance to cardiac function (de Lucia et al., 2018), appear to have a spatial localization trend within the RAGP (Figures S5B–S5D). Modules 4 and 6 delineate the separation between states B and C where module 4 is characterized by high expression in states B and D and includes a variety of receptor subtypes. Module 6, consisting of a variety of neuropeptides and neuropeptide receptors, has a broader range of expression across all samples, with mixed expression in most states but particularly high expression in state C. Genes in module 1 were upregulated in states D and F and included neuropeptides such as *Npff* and *Nppa* as well as a voltage-gated potassium channel, *Kcnip1* (Figure 4F). Examination of ion channels and glutamatergic and GABAergic receptors also show a range of expression across the identified transcriptional states (Figures S4B and S4C).

### Correlated cholinergic and catecholaminergic gene expression in RAGP neurons

**Figure 5:**
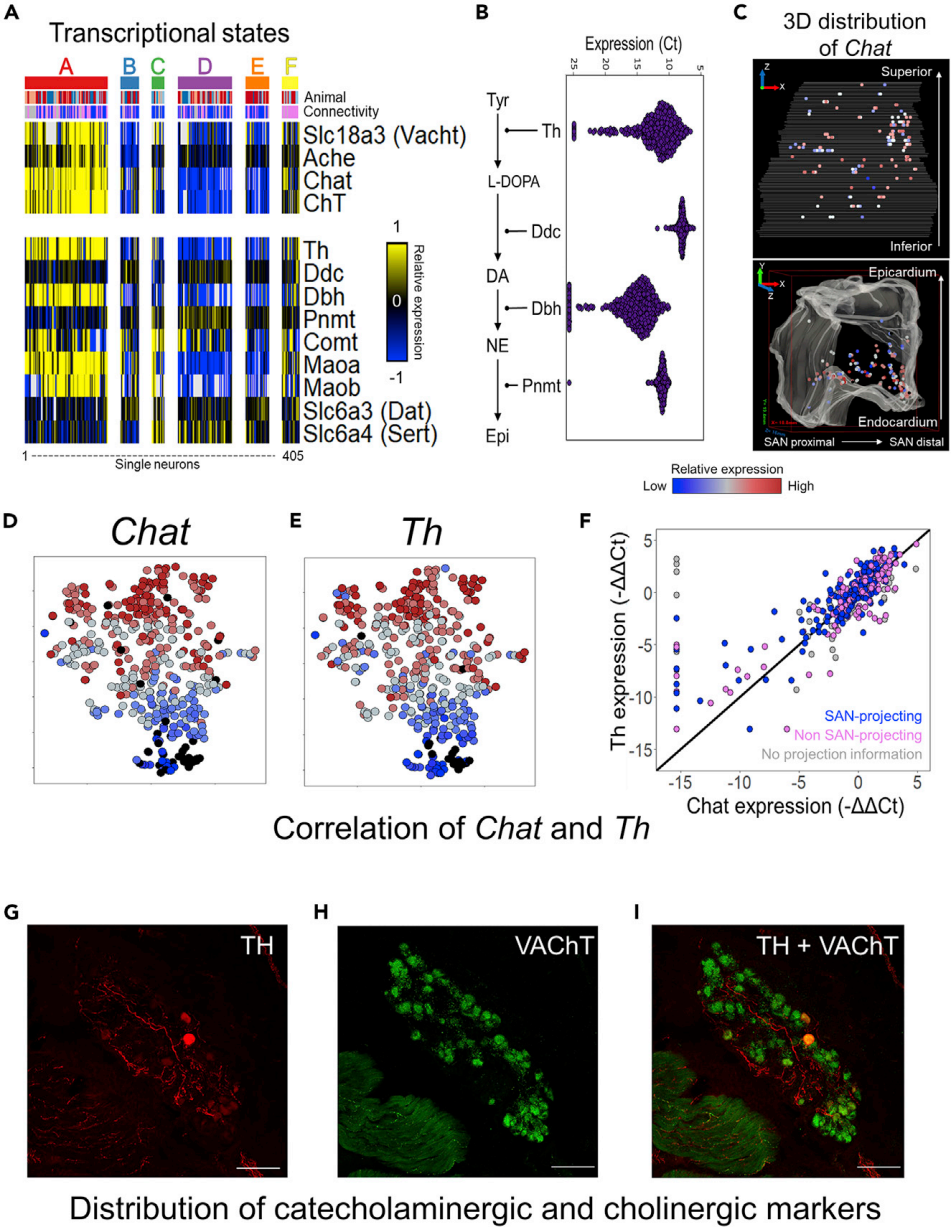
Correlated cholinergic and catecholaminergic gene expression in RAGP neurons (A) Transcriptional state-wise gene expression of the components of acetylcholine and catecholamine biosynthesis and transport processes, across 405 single neurons assayed through HT-qPCR in RAGP. (B) Beeswarm plot showing the abundance and the range of expression of key genes involved in catecholamine biosynthesis across 405 single RAGP neurons from n = 4 animals. (C) Visualization of *Chat* gene expression within the 3D anatomical framework for a representative RAGP. The bounding box on the lower panel shows 18.8 mm, 19.4 mm, and 16 mm on the x, y, and z axis, respectively. (D and E) The distributions of *Chat* and *Th* gene expression overlap within the transcriptional landscape as visualized in the tSNE plots. (F) Correlated gene expression of *Chat* and *Th* across single neurons in RAGP (R^2^ = 0.69, pvalue < 2.2 × 10^−16^). The pairwise comparison of gene expression levels is shown for SAN-projecting and non SAN-projecting neurons (n = 4 animals). The points marked gray correspond to RAGP neurons without information on SAN projection, as these were microdissected from a pig heart without a tracer injection into the SAN region. (G–I) Confocal images showing a cluster of neurons within RAGP double stained for TH (G) and VAChT (H). Colocalization of TH and VAChT in a subset of neurons (I).

Examination of genes involved in acetylcholine and catecholamine biosynthesis and transport processes shows consistent expression across all RAGP and between SAN-projecting and non SAN-projecting neurons and also reveal a surprising level of co-expression (Figures 5A, 5B, S2C, and S6A–S6D). A closer look at the genes involved in the catecholamine biosynthesis showed that *Th*, the rate-limiting enzyme in the production of all catecholamines, and *Dbh*, responsible for the conversion of dopamine to norepinephrine are expressed over a wide range and show a high degree of coexpression (Figures 5B andS6B). *Ddc*, the enzyme converting L-DOPA to dopamine, and *Pnmt*, responsible for the conversion of norepinephrine to epinephrine, were both stably expressed in the majority of samples as seen by the high abundance and narrow range of expression for both genes (Figure 5B). This further underscores the regulation of catecholamine biosynthesis process at the level of *Th* and *Dbh* as the rate-limiting enzymatic steps whose gene expression levels are highly variable across single neurons. Notably, *Nos1* and *Vip*, both known to be expressed in certain cholinergic neurons (Blottner and Baumgarten, 1992; Schotzinger et al., 1994) showed no correlation of expression to *Chat* in the RAGP (Figures S7A and S7B). Expression of *Vip* and *Nos1* was detectable in nearly all the assayed neurons (Figures S7C and S7D). Additionally, *Ret* and *Ntrk1,* that showed differential expression between SAN-projecting and non SAN-projecting RAGP neurons, showed similar patterns of co-expression with the cholinergic and catecholaminergic markers (Figure S3, Module 2).

Visualization of key genes such as *Chat* and *Th* within the 3D anatomical framework of a representative RAGP revealed that their wide range of expression is distributed spatially throughout the RAGP (Figures 5C and S8A, Video S3). Visualization of the expression distribution of *Chat* and *Th* through a tSNE map revealed distinct and overlapping gradients within the transcriptional landscape (Figures 5D and 5E). Single-cell scale RNA-seq data suggested regional co-expression of *Th* and *Chat* (Figure 2F). Building on these results, single neuron gene expression analysis showed that *Chat* and *Th* were highly correlated across single neurons in the RAGP (Figure 5F). Interestingly, gene co-expression of these cholinergic and catecholaminergic markers was in stark contrast to protein expression patterns that showed much reduced overlap of expression between TH and VAChT, another cholinergic marker, in individual neurons (Figure S6C). Immunohistochemistry for TH and VAChT revealed that a majority of neurons showed robust protein expression of VAChT with a subset co-staining for TH (Figures 5G–5I). A more detailed quantitative analysis of TH and VAChT protein co-expression are included in a complementary study (Hanna et al., 2021). This finding suggests that post-transcriptional regulation plays a key role in shaping the neurotransmitter patterns within RAGP. In particular, the high correlation between *Th* and *Chat* at the mRNA level suggests that the RAGP neurons are poised to use both cholinergic and catecholaminergic processes, along the lines of multipotential neuronal phenotypes observed in CNS (Park et al., 2014).

Video S3. 3D spatial distribution of Chat and Th expression across a representative RAGP, related to Figure 5

### Neuropeptidergic interaction networks in the pig RAGP

**Figure 6:**
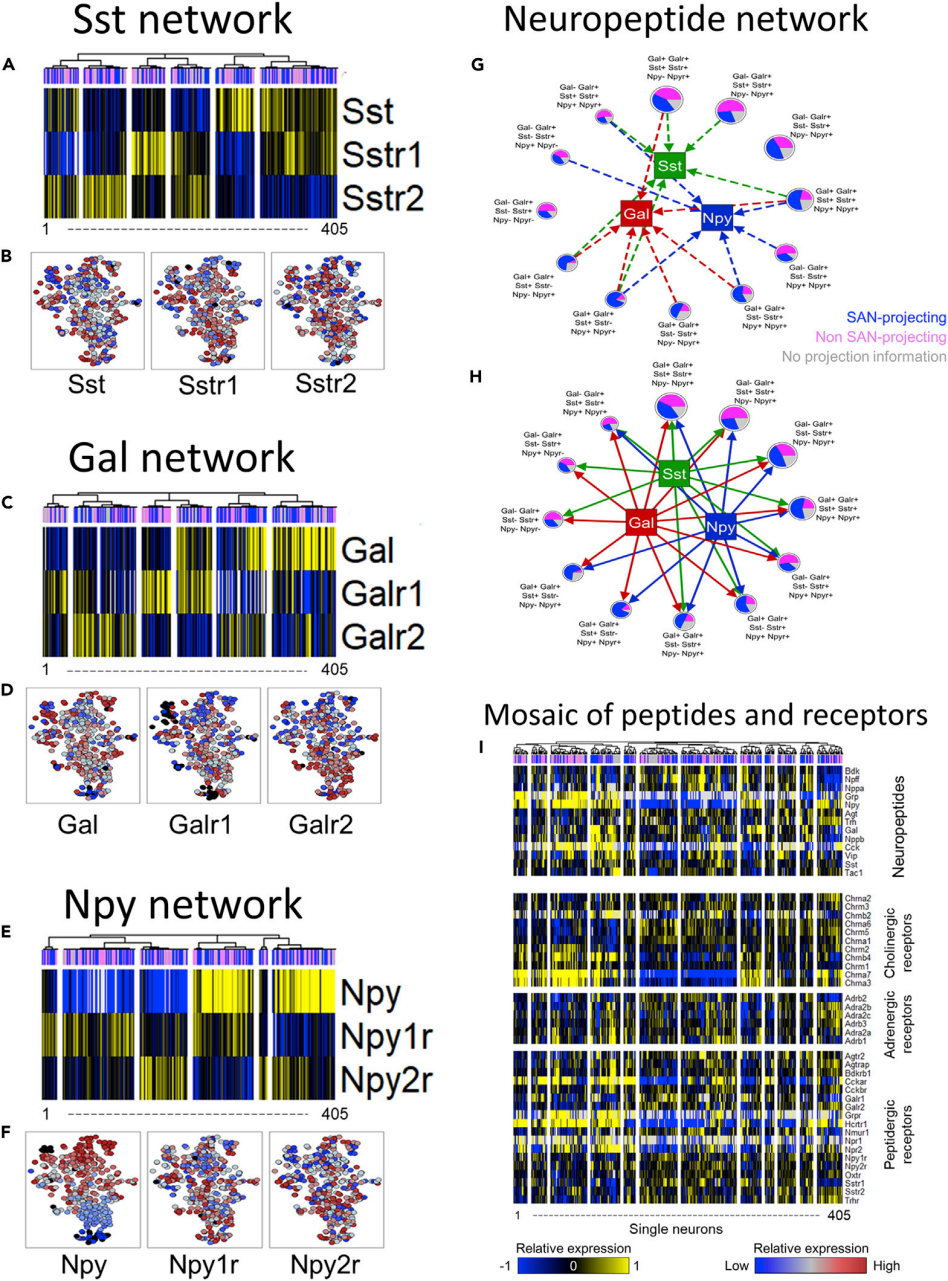
Neuropeptidergic interaction networks in the pig RAGP (A,C, and E) Expression patterns of somatostatin (A), galanin (C), and neuropeptide Y (E) and their cognate receptors across 405 single RAGP neurons (n = 4 animals) assayed through HT-qPCR. (B,D, and F) The transcriptional landscape across all RAGP colored for expression of the neuropeptides and their cognate receptors. (G,H) Interaction networks of neuronal subtypes defined based on the combinatorial pattern of neuropeptides and their receptor expression. (G) The interaction network subset corresponding to the neuronal subtypes producing the neuropeptides somatostatin (*Sst*), galanin (*Gal*), and neuropeptide Y (*Npy*). The circular nodes denote the neuronal subtypes.The size of the node is proportional to the number of single neurons belonging to each subtype. The pie chart within each circular node indicates the proportion of the neurons within that subtype that are identified as projecting to the SAN region. The arrows from the circular nodes denoting the neuronal subtypes connect to the square-shaped nodes denoting the three neuropeptides, based on which subtypes show the corresponding neuropeptide gene expression above a specified threshold. The color of the arrows matches the color of the corresponding target square-shaped node. (H) The interaction network subset corresponding to the neuropeptide receptor expression across the neuronal subtypes. The notation of circular and square-shaped nodes is the same as in pane (G). The arrows connect each neuropeptide to the neuronal subtypes based on which subtypes express any the corresponding neuropeptide receptors above a specified threshold. The color of the arrows corresponds to the color of the neuropeptide node. (I) Combinatorial pattern of expression of a wide range of neuropeptides and receptors of neurotransmitters across single neurons in RAGP (n = 4 animals).

We examined the co-expression patterns of neuropeptides and their cognate receptors to identify putative local paracrine networks within RAGP. Identifying neuronal subsets based on co-expression patterns of a neuropeptide with its receptors allows us to classify groups of neurons that exhibit autocrine or paracrine signaling. We examined local paracrine networks for three important neuropeptides, *Sst, Gal,* and *Npy,* in detail (Figures 6A–6F, S8B–S8D, and S9).

In the case of somatostatin and its receptors, *Sstr1* and *Sstr2*, neurons cluster into six groups based on the presence or absence of each gene (Figure 6A). This revealed one neuronal subset that expresses somatostatin, but not its receptors, and therefore can transmit but not respond to the somatostatin signal in the RAGP network. Meanwhile, two neuronal subsets were positive for somatostatin as well as either receptor *Sstr1* or *Sstr2*, representing groups that can both transmit and be activated by somatostatin.The remaining three neuronal subsets do not synthesize but can respond to somatostatin (Figures S9A and S9D). Similar to *Sst, Gal* and *Npy* and their cognate receptors each clustered into six neuronal subsets. When categorizing each neuronal subset based on the presence or absence of each gene, however, the six neuronal subsets were reduced to 5 categories for the *Gal/Galr1/Galr2* set and 4 identifiers for the *Npy/Npy1r/Npy2r* set (Figures 6C, 6E, S9B, S9C, S9E, and S9F).

Despite the strong patterns of expression seen within each individual network, none of the genes in either the *Sst/Sstr1/Sstr2* or *Gal/Galr1/Galr2* set showed a discernible gradient across the broader transcriptomic landscape (Figures 6B, 6D, and 6F). However, *Npy* (and not its receptors) showed a gradient of expression that largely mirrored the *Th* and *Chat* gradients, consistent with the co-expression of these genes across single neurons (Figure 6F). Taken together, the results indicate that while *Sst, Gal*, and *Npy* each have distinct co-expression patterns that outline local paracrine networks, the signaling of any one neuropeptide alone is unlikely to be the main driver of transcriptional states of RAGP neurons.

Integrating all three paracrine networks at once revealed that the overall combination is more complex than any individual neuropeptide driven network (Figures 6G, 6H, and S9). Each neuron was categorized as neuropeptide +ve or neuropeptide -ve, receptor +ve or receptor -ve, based on whether the gene expression of the given peptide/receptor was above the median expression value for that gene across all the neurons. Applying such a categorization to the 405 laser capture microdissected RAGP neurons resulted in 61 distinct neuronal categories (out of a possible set of 64), indicating a combinatorial network of neuropeptide signaling (Data S1). Of these 61 categorized neuronal states, a set of 12 states consisted of more than 10 sampled neurons each, representing a total of 224 out of the 405 single neurons (about 55%). A network representation of these 12 neuronal subsets highlighted the putative autocrine and paracrine network of neuropeptide signaling between RAGP neurons (Figures 6G and 6H). The conceptual framework outlined by this paracrine network of neuromodulatory factors offers insight into different approaches for pharmacological interventions in cardiovascular disease. Additionally, visualization of these individual networks within their 3D anatomical framework also suggests the potential for widespread signaling throughout the RAGP and enables a much more selective and specific study of the physiological involvement of these neuronal clusters (Figures S8B–S8E, Video S4). Further expanding the network analysis to include all assayed neuropeptides and receptors revealed a combinatorial mosaic of neuropeptide/receptor expression (Figure 6I), indicating a complex local neuromodulatory network within RAGP.

Video S4. Neuropeptidergic interaction networks within the 3D anatomical framework of a representative RAGP, related to Figure 6

## Discussion

In this study, we performed a combination of spatially-tracked single-cell transcriptomic analysis of an intrinsic cardiac ganglion. Our results uncovered the complex molecular landscape and paracrine networks of RAGP neurons in the pig heart. RNA-seq analysis revealed that the composition of RAGP neurons is distinct from that of neuronal subtypes in the CNS, with minimal overlap in the combinatorial expression patterns of neuronally enriched genes. Using HT-qPCR of hundreds of single RAGP neurons, we identified neuronal transcriptional states with distinct gradients across the transcriptomic landscape. A remarkable finding was a high prevalence of coexpression of cholinergic and catecholaminergic neuronal markers *Chat* and *Th* in a large fraction of the RAGP neurons, contrary to immunohistochemistry data showing only minimal coexpression, suggesting multipotential phenotypes. The gene expression profiles of SAN-projecting RAGP neurons were distributed across multiple neuronal states without a single gene or gene expression module serving as an exclusive marker of RAGP neuronal connectivity to the SAN region. Our integrative analysis revealed complex expression patterns of neuropeptide signaling indicating that any individual neuropeptidergic system is unlikely to act as the main driver of neuronal transcriptional states. Neuromodulation of vagal activity to regulate cardiac function is an active and growing research area with much potential for transforming clinical care in heart disease. The single neuron transcriptomic landscape and paracrine networks uncovered in this study form the foundation for developing new neuromodulatory targets for improving heart health.

In recent years, the single-cell RNA-Seq studies of neurons isolated from various CNS components show distinct separations along the transcriptomic landscapes with individual clusters specifically linked to excitatory or inhibitory processes (Chen et al., 2019; Darmanis et al., 2015; Hu et al., 2017; Kupari et al., 2019; Li et al., 2020). Given the complex neural networks to and from the CNS that influence the ICNS, we compared our RNA-Seq data with RNA-Seq data from single CNS and PNS neurons to compare cell types across central and peripheral neurons.

Interestingly, our data showed little to no alignment with the cell populations identified in CNS neurons. In contrast with CNS neurons which cluster distinctly into dedicated phenotypes, neurons of the RAGP resemble mixed neuronal types with a gradient of expression as represented by a single cloud across the transcriptomic landscape (Figures 2C and 4B) (Hu et al., 2017; Kupari et al., 2019). These results suggest that comparing RAGP to CNS neuronal types is not akin to comparing the variability of neural phenotypes in a single brain nucleus to the CNS as a whole. While transcriptomics and proteomics with 3D anatomical location tracking have been established for the brain (Allen Brain Atlas) (Goel et al., 2014; Hawrylycz et al., 2012; Roth et al., 2006; Shen et al., 2012), to our knowledge, we are the first group to attempt this at the mammalian heart (Achanta et al., 2020). This work enabled creation of a 3D model of pig RAGP that precisely integrates molecular data of single neurons into the 3D anatomical framework. Unlike the CNS, where molecular profiles correspond to specific anatomically located nuclei, we have found no discernable connection between the molecular profiles and anatomical locations within the RAGP (Goel et al., 2014; Hawrylycz et al., 2012; Roth et al., 2006; Shen et al., 2012). This suggests that the physiological functions attributed to the ICNS are likely to not be restricted to a particular anatomical location within any ganglionic plexus, and that the RAGP, and possibly other ganglionic plexuses in the ICNS, is composed of a combination of neuronal types whose integrative control of the heart enables the complex response patterns observed in physiological studies (Hamon et al., 2017).

Available physiological data suggest the notion that RAGP may consist of 80% locally connecting neurons, with the remainder distributed between those receiving afferent and motor input (both parasympathetic and sympathetic) (Ardell et al., 2015; Armour, 2008; Hoard et al., 2008; Hopkins and Armour, 1984; Kawashima, 2005; Neel and Parsons, 1986). Our results showed that the underlying transcriptomically-defined molecular states of neurons in RAGP are not necessarily aligned with the phenotypic categories postulated by physiological findings. We identified six transcriptional states of RAGP neurons that are distinguished by combinatorial patterns of gene expression modules that span several neuronal functions/phenotype categories, including cholinergic, adrenergic, neurotransmitter, receptor expressions and ion channels. No state was exclusively represented by individual gene expression modules or a subset of the genes. Such combinatorial patterning of neuronal states was present similarly in SAN-projecting and non SAN-projecting neurons. Analysis of 3D spatial location of connectivity-based (SAN projecting, non SAN-projecting) and transcriptional state-based neuronal groups showed a heterogeneous distribution across the anatomical and transcriptional landscape of the RAGP. Our results on the combinatorially organized gene expression modules defining the spatially distributed RAGP neuronal states can serve to explain the variability of physiological responses observed in experimental disruption of ICNS circuits (Berger et al., 2019; Driessen et al., 2016; Qin et al., 2017).

Distinct neuron types that are sympathetic or parasympathetic have been identified in the ICNS in various immuno-histochemical studies for ChAT and TH to identify cholinergic or adrenergic phenotypes, respectively (Hoard et al., 2008; Hoover et al., 2009; Horackova et al., 2000; Richardson et al., 2003; Rysevaite et al., 2011), where the majority have found the neurons to be either parasympathetic or sympathetic in nature. However, some have found that ICNS neurons are not exclusively parasympathetic or sympathetic, with reports showing 10-20% of the cardiac GP co-expressing both ChAT and TH (Rysevaite et al., 2011; Zarzoso et al., 2013).

One of the more significant findings of this study is the co-expression of cholinergic and catecholaminergic pathway genes in our data (Figure 5). To our knowledge, this is the first study to report co-expression of both *Chat* and *Th* in the RAGP, with 90–95% of neurons showing detectable expression of both genes, and 76% of neurons showing abundant expression of both genes at the single-cell transcriptional level (Figures 5A–5H). Of note, the ICNS is comprised of two neuronal types, large principle neurons (PNs) and small intensely fluorescent (sif) cells, the latter of which contain a wide range of neurotransmitters and are often more likely to express adrenergic phenotypes (Hall and Landis, 1991; Rysevaite et al., 2011; Wake and Brack, 2016). Based on neuron size and through the use of LCM, we specifically targeted our single-cell sampling to PNs (Figure 3C). While it is possible that some sif cells were collected along with the PNs, we believe that the data and therefore the coexpression patterns between *Chat* and *Th* can be attributed mainly to the PNs in the RAGP. Testing the protein level expression of these enzymes in the RAGP showed consistent results with previous studies in the ICNS. Confocal microscopy studies of RAGP showed that 99% neurons were VAChT positive and 10-20% neurons were also positive for TH (Figures 5G–5I). We interpret these differences between gene and protein expression levels as representative of multipotential phenotypes that are observed at the transcriptional scale that are then shaped further post-transcriptionally to yield a specific distribution in a given physiological context.

Further evidence of post-transcriptional modifications is the strong co-expression patterns between *Chat*, *Th*, and genes known to differentiate between neuronal subtypes such as *Ret* and *Ntrk1* in other sympathetic ganglia (Furlan et al., 2016). *Ret* and *Ntrk1* encode for neurotrophic factor receptors that bind glial-derived neurotrophic factor (GDNF) and nerve growth factor (NGF), respectively. Interestingly, previous experiments have shown that cardiac neurons are immunoreactive for TrkA (encoded by *Ntrk1*) regardless of whether or not they are immunoreactive for TH (Hoard et al., 2008). We and others have shown that cells retain their plasticity beyond development and are able to adapt to perturbation based on the inputs they receive in normal and diseased states by transcriptional, as well as post-transcriptional regulation (Dulcis et al., 2013; Park et al., 2014). For example, we have previously shown that TH immunoreactive neurons in the brainstem are organized along a gradient of catecholaminergic (*Th+/Fos-*) and non-catecholaminergic (*Th-/Fos+*) neuronal states (Park et al., 2014), and this gradient shifts in response to physiological perturbation such as sustained hypertension. Our results on correlation of *Th* and *Chat* raise an intriguing possibility of such plastic adaptive dynamics within the RAGP, with functional effects on vagal control of cardiac function.

We delved into three peptidergic systems, *Gal, Sst,* and *Npy*, which displayed distinctive coexpression patterns with their respective receptors. These neuromodulators have been shown to have important effects on cardiac function and vagal tone (Beal and Martin, 1986; Gorky and Schwaber, 2019; Habecker et al., 2016; Herring and Paterson, 2009; Herring et al., 2012; Hökfelt et al., 1977; Moriarty et al., 1992; Smith-White et al., 2003). High concentrations of *Sst* have been found in cardiac tissues, specifically in the right atrium and the atrioventricular node, where specialized conducting and pacemaker cells are found (Day et al., 1985). *Sst* is found mostly in GABAergic neurons and mediates antagonism of sympathetic processes on a broad scale as well as promotes parasympathetic effects, specifically reducing cardiac contractility in an Ach-dependent manner (Gorky and Schwaber, 2019). Meanwhile both *Gal* and *Npy* have been identified as co-transmitters in adrenergic neurons and have been shown to work in similar and complementary manners with respect to vagal control (Herring et al., 2012). While *Gal* has been shown to attenuate cardiac vagal activity with no effect on blood pressure, *Npy* does just the opposite, increasing blood pressure but having no inhibitory effects on vagal activity (Smith-White et al., 2003). Studies have shown that *Gal* and *Npy* released from sympathetic neurons inhibit the release of acetylcholine in the cardiac cholinergic postsynaptic neurons (Habecker et al., 2005). Our results on the expression of *Sst*, *Gal*, and *Npy* in the RAGP neurons suggest a strong likelihood of these neuromodulatory interactions arising from within the RAGP paracrine networks to mediate parasympathetic and sympathetic control of cardiac function. This data is largely consistent with our previous findings in the rat heart that also show a wide range of expression and combinatorial patterns between peptides and their cognate receptors (Achanta et al., 2020). We examined each of these peptidergic networks separately and in combination with one another in the larger context of the neuronal network. While examination of one neuropeptide-receptor set at a time reveals discrete and overlapping co-expression patterns, these gradients are absent from the broader transcriptomic landscape, further underscoring that examination of one peptidergic network is insufficient to gain an understanding of the network as a whole. Combination of the three peptidergic networks yielded a wide range of combinatorial gene expression patterns and reveals a widespread signaling network where almost all sampled neurons are capable of being activated by one or more neuropeptides. Notably, *Gal* and *Npy,* which have been shown to be released together in sympathetic neurons of the stellate ganglia (Habecker et al., 2005), showed partly overlapping gene expression patterns across RAGP neurons, suggesting distinctive adaptive and neuromodulatory phenotypes in the RAGP versus elsewhere.

Expanding the network analysis to account for the wide range of neuropeptide systems expressed within RAGP suggests a conceptual formulation of RAGP as a highly adaptive and dynamic system driven by combinatorial patterns of neuromodulators acting in local paracrine networks. The dynamicism observed in these paracrine networks along with the level of correlation between cholinergic and catecholaminergic markers underscores the distinct composition of the ICN in comparison with the central nervous system and other studied ganglia (Furlan et al., 2016; Hu et al., 2017; Zeisel et al., 2018). This study lays the groundwork for future studies to further illuminate how the unique organization of the ICN is crucial to the pacemaking activities of the SAN and cardiac function. The neural network of the ICN is aptly referred to as the “little brain” with its high level of complexity and large percentage of LCNs that are capable of remodeling upon cardiac disease and injury (Armour, 2008; Rajendran et al., 2016). We speculate that the complexity and variability associated with such a wide range of cardiac functions may simply require more precise and dynamic control than other autonomic ganglia. The wide diversity of potential transmitters may therefore be necessary in order to meet the needs resulting from such a diversity of functions (Habecker et al., 2016).

Our data demonstrates that the local cardiac ganglia harbor anatomical and molecular features necessary to function as complex signal processing units that critically mediate vagal control of heart function and health. These conceptual advances provide the necessary anatomical and molecular foundation for new functional and translational research. In addition, our study provides the data needed to begin a modeling effort focused on neuroanatomical and electrophysiological properties of the intrinsic cardiac neurons. Integrating these findings with other data obtained from our collaborative SPARC consortium will enable development of computational models of neurons and networks in the RAGP as a critical mediator of vagal control of the heart (Hanna et al., 2021). With the RAGP as a starting point, the RAGP network model can be further expanded into a systems model of autonomic control of cardiovascular function to test novel control strategies for neuromodulation and to open the door to more effective therapeutics.

### Limitations of the study

• A FastBlue retrograde tracer was injected into the SA-node of the pig to label SAN-projecting neurons in the RAGP. It is possible that there was incomplete penetration of the tracer and that some neurons identified as non SAN-projecting due to lack of FastBlue signal indeed project to the SA-node.
• The present findings are true for one species, the Yucatan minipig, and it should be investigated how these results are represented across species, particularly in humans
• The relative homogeneity of the transcriptional data throughout the RAGP suggests that different cardiac regulatory functions may not be localized to distinct subpopulations but are rather distributed throughout, which should be further investigated. If this is true, it may be that there is local coordination for the regulation of different cardiac functions and regions that are integrated specifically in different populations of the ICN.
• The present study investigates the healthy animal, and while there are implications for disease, the exact manifestations of these networks in the disease state remain to be explored.

## STAR★Methods

### Key resources table

**Table:** 

REAGENT or RESOURCE	SOURCE	IDENTIFIER
**Antibodies**		

anti-VACht antibody	Synaptic Systems	Cat. No. 139103; RRID: AB_887864
anti-TH antibody	Millipore	Cat. No. AB1542; RRID:AB_90755

**Critical commercial assays**		

SuperScript VILO Master Mix	Thermo Fisher Scientific	Cat#:11756500

**Deposited data**		

Genotype-Tissue Expression (GTEx) Portal	GTEx Analyis V8	dbGaP Accession phs000424.v8.p2; https://www.ncbi.nlm.nih.gov/projects/gap/cgi-bin/study.cgi?study_id=phs000424.v8.p2
Single-nucleus RNA sequencing analysis of adult mouse cerebral cortex	Hu et al.2017	GSE106678
Single cell data from the human CNS	Allen Brain Map	RRID:SCR_017001; https://portal.brain-map.org/atlases-and-data/rnaseq/human-multiple-cortical-areas-smart-seq
RNAseq comparisons to the peripheral nervous system	Zeisel et al.2018	http://mousebrain.org/genesearch.html; Mousebrain.org.level1/L1_Enteric.loom; Mousebrain.org.level1/L1_Sympathetic.loom
RNAseq comparisons to the peripheral nervous system	Furlan et al.2016	http://linnarssonlab.org/sympathetic/
Raw sequencing data generated in this study	GEO database	GSE154119; GEO SuperSeries GSE154411
Raw and processed HT-qPCR data of single neurons from the pig RAGP	GEO database	GSE149212; GEO SuperSeries GSE154411
Acquisition of single neurons and regional neuronal samples from the porcine Right Atrial Ganglionic Plexus (RAGP) through Laser Capture Miscrodissection	Stimulating Peripheral Activity to Relieve Conditions (SPARC) data portal, sparc.science	https://doi.org/10.26275/56h4-ypua
Spatially Tracked Single-Cell-Scale RNAseq of Porcine Right Atrial Ganglionic Plexus (RAGP) Neurons	Stimulating Peripheral Activity to Relieve Conditions (SPARC) data portal, sparc.science	https://doi.org/10.26275/kabb-mkvu
Transcriptional Diversity of Single Neurons in the Porcine Right Atrial Ganglionic Plexus (RAGP)	Stimulating Peripheral Activity to Relieve Conditions (SPARC) data portal, sparc.science	https://doi.org/10.26275/5jki-b4er
Spatially Tracked Single-Neuron Transcriptomics of a Female Porcine Right Atrial Ganglionic Plexus (RAGP)	Stimulating Peripheral Activity to Relieve Conditions (SPARC) data portal, sparc.science	https://doi.org/10.26275/qkzi-b1mq
Spatially Tracked Single-Neuron Transcriptomics of a Male Porcine Right Atrial Ganglionic Plexus (RAGP)	Stimulating Peripheral Activity to Relieve Conditions (SPARC) data portal, sparc.science	https://doi.org/10.26275/255m-00nj
Dataset containing figures from the paper as well as code and necessary files to generate them.	Stimulating Peripheral Activity to Relieve Conditions (SPARC) data portal, sparc.science	https://doi.org/10.26275/jdws-d7md

**Experimental models: organisms/strains**		

Model organism: Yucatan minipig	S&S Farms	N/A

**Software and algorithms**		

R version 3.6.3 (Holding the Windsock)	The R Foundation for Statistical Computing	https://www.r-project.org/
Subread R package	Liao et al., 2019	https://bioconductor.org/packages/release/bioc/html/Rsubread.html
ComBat-seq	Zhang et al., 2020	*sva* package; https://bioconductor.org/packages/release/bioc/html/sva.html
DESeq2	Love et al., 2014	https://bioconductor.org/packages/release/bioc/html/DESeq.html
SCnorm	Bacher et al., 2017	https://bioconductor.org/packages/release/bioc/html/SCnorm.html
Real-Time PCR Analysis Software	Fluidigm	https://www.fluidigm.com/software
NormqPCR	Bioconductor	RRID:SCR_003388; https://bioconductor.org/packages/release/bioc/html/NormqPCR.html
cluster R package	Comprehensive R Archive Network (CRAN)	https://cran.r-project.org/web/packages/cluster/index.html

### Resource availability

#### Lead contact

Further information and requests for resources should be directed to and will be fulfilled by the Lead Contact, Rajanikanth Vadigepalli: rajanikanth.vadigepalli@jefferson.edu.

#### Material availability

This study did not generate new unique reagents.

#### Data and code availability

The authors declare that all the data supporting the findings of this study are available within the article and its supplemental information files or from the corresponding author upon reasonable request. Raw sequencing data generated in this study have been deposited at the GEO database under accession code:GSE154119. Raw and processed HT-qPCR data of single neurons from the pig RAGP have been deposited in the GEO database under accession code: GSE149212. The RNAseq and HT-qPCR datasets constitute a GEO SuperSeries GSE154411. All sample acquisition images, raw and processed transcriptomic data, and annotations pertaining to 3D spatial location are publicly available in the sparc.science (RRID:SCR_017041) repository with the digital object identifiers https://doi.org/10.26275/56h4-ypua, https://doi.org/10.26275/kabb-mkvu, https://doi.org/10.26275/5jki-b4er, https://doi.org/10.26275/qkzi-b1mq, and https://doi.org/10.26275/255m-00nj. A dataset containing high-resolution figures, supplemental figures, movies and files, as well as the TissueMapper XML annotations and the R code to generate the data-driven plots and visualizations illustrated in the figures are available at https://doi.org/10.26275/jdws-d7md. Data analyzed from Hu et al. (2017) is available in the GEO database under the accession code GSE106678. Data from GTEx was retrieved from GTEx Analysis V8 (dbGaP Accession phs000424.v8.p2 https://www.ncbi.nlm.nih.gov/projects/gap/cgi-bin/study.cgi?study_id=phs000424.v8.p2). Single cell data from the Human CNS was obtained through the Allen Brain Map (RRID:SCR_017001) project and is available at https://portal.brain-map.org/atlases-and-data/rnaseq/human-multiple-cortical-areas-smart-seq. Data for RNAseq comparisons to the peripheral nervous system is available through online resources associated with Zeisel et al. (2018) (http://mousebrain.org/genesearch.html; Mousebrain.org.level1/L1_Enteric.loom; Mousebrain.org.level1/L1_Sympathetic.loom) and Furlan et al. (2016) (http://linnarssonlab.org/sympathetic/).

### Experimental model and subject details

Animal experiments were performed in accordance with the UCLA Institutional Animal Care and Use Committee, and euthanasia protocols conform to the National Institutes of Health's Guide for the Care and Use of Laboratory Animals (2011). Data for all experiments were collected from 2 male and 2 female normal healthy Yucatán minipigs(>3 months old).

### Method details

#### Optimization of neural tracers for laser capture microdissection

Fast Blue (Polysciences, 17740-1), CM-DiI (Thermo Fisher, C7000), Fast DiI (Thermo Fisher, D3899), TMR-Dextran (Thermo Fisher, D3308), FluoroGold (Fluorochrome) as candidate fluorescent tracers were scanned for their compatibility with the dehydration protocol of laser capture microdissection before the tracer was adapted in the minipig. In each animal, one of the five tracers was injected into Sprague Dawley rats (3-4 months old, purchased from Envigo) sino-atrial node at the same volume of 10 μL and the heart tissues were harvested in the time window 10 am-12 pm 14 days after injection and stored in OCT immediately. Cryosections were visualized under a fluorescence microscope before and after the dehydration steps necessary for laser capture microdissection and acquisition of single neuron samples (as detailed below). All five tracers labeled intrinsic cardiac nervous system neurons successfully but only CM-DiI and Fast Blue-labeled neurons remained intact on the heart sections without fixation, whereas TMR-Dextran and FluoroGold-stained neurons were not visible under the microscope under the conditions suitable for laser capture microdissection. Only Fast Blue provided reliable and consistent labeling visible under laser capture microdissection microscope after the necessary dehydration procedure and was used in the subsequent tracing experiments in the study.

#### Neural tracing experiments

For initial surgery, following sedation (induction: ketamine (10mg Kg-^1^ IM)/midazolam (1mg Kg-^1^ IM), maintenance: isoflurane 1-2% inhalation) and intubation, a right unilateral thoracotomy was performed by dividing the pectoral muscle, making a small incision in the pericardium, and exposing the right atrial-superior vena cava junction. Then 5mg of Fast Blue (Polysciences), a neuronal tracer that is retained and diffuses within the lipid bilayer, in 250uL of sterile water (2% weight/volume) was injected using a 27-gauge needle into the SAN region. A chest tube was placed, and the incision was closed. Immediately prior to removal, the chest tube was aspirated. Tissues were harvested in a terminal procedure at least 3 weeks later as described below.

#### Porcine tissue collection

Following sedation (induction: tiletamine-zolazepam 6mg Kg^-1^ IM, maintenance: isoflurane 1-2% inhalation) and intubation, we performed a midline sternotomy and exposed the heart. A heparin bolus of 5000U IV was administered, and the pig was then placed in ventricular fibrillation with application of a 9V battery to the surface of the heart. The heart was explanted and syringe-flushed with heparinized normal saline (5U mL^-1^) via the transected aorta. The area of interest (RAGP-SAN region) was then excised and rinsed in heparinized saline. RAGPs were separated from the SANs, immersed in 1x PBS at RT for 30 seconds and transferred to 25% Optimal Cutting Temperature compound (OCT, TissueTek; VWR 25608-930), followed by 50% OCT and then embedded in 100% OCT and placed in a cryomold. The cryomold was placed in a methanol dry ice bath for flash freezing.

#### Cryosectioning and staining

RAGP was sectioned along the superior-inferior axis (corresponding to the source animal) at 40μm thickness, yielding between 447-1,030 sections per RAGP, with corresponding blockface images. Tissue sections were mounted on PPS membrane slides (Leica Microsystems, Catalog 11600294). Slides were fixed in icecold ethanol (100% Ethanol) for a minute followed by four minutes of staining with 0.0001% Cresyl Violet (ACROS Organics, AC229630050). The slides were dehydrated using 95% and 100% Ethanol followed by Xylene for one minute each. The staining protocol was kept to under 15 minutes and an RNAse inhibitor (Invitrogen SUPERase-In RNase Inhibitor, Catalog AM2696) was added to all aqueous reagents to preserve RNA quality. More detailed methods can be found in the research protocol available at https://doi.org/10.21203/rs.3.pex-928/v1.

#### Laser capture microdissection

Slides were stained and immediately processed for sample collection to preserve RNA quality using Laser Capture Microdissection (Arcturus, ThermoFisher). SAN projecting and non SAN-projecting neurons were identified under fluorescence using FastBlue (excitation 365 nm, emission 420 nm) and 0.0001% cresyl-violet (585 nm excitation and 627 nm emission) stain and only cresyl-violet stain respectively. While it is possible that the tracer failed to properly label all SAN-projecting neurons, for the purposes of this analysis, all cells without FastBlue labeling were referred to as “non SAN-projecting” neurons. Neurons collected for RNAseq were identified using 0.1% cresyl-violet stain. Samples were collected on LCM Caps and stored at -80°C. Samples were lysed at the time of gene expression experiments with appropriate lysis reagents based on the downstream processing protocols (RNASeq or HT-qRTPCR). We stochastically sampled nearly all of the neuronal clusters encountered throughout the RAGP from one of the animals, while the other three animals were stochastically sampled throughout the RAGP in a less extensive manner. More detailed methods can be found in the research protocol available at https://doi.org/10.21203/rs.3.pex-927/v1.

#### Mapping LCM samples onto the reconstructed 3D stack

2D images of tissue blockface were acquired after each section. Using Tissue Mapper software 3D volume of each RAGP was created from the outer contours of blockface images. Images taken during LCM sample collection were assigned to corresponding sections in the 3D stack. Anatomical features and neurons (collected and not collected) were assigned markers enabling extraction of specific XYZ coordinates for the neurons in RAGP. Blockface images and acquisition images can be found at https://doi.org/10.26275/56h4-ypua. More detailed methods can be found in the research protocol available at https://doi.org/10.21203/rs.3.pex-922/v1.

#### RNASeq library preparation

Samples on LCM caps were processed for single cell scale RNASeq based on a protocol modified from Foley et al., 2019(Foley et al., 2019). We followed the steps recommended for fresh frozen tissue with the following modifications: We used 10μl of lysis buffer on HS Caps (original protocol recommends 5 μl). In the step 3 for performing PCR amplification of the tagged mRNA fragments, we used 22 cycles of PCR (maximum allowed). The original protocol relies on a formula to compute amplification cycles, and suggested 18 cycles based on sample RNA input and number of samples multiplexed per RNASeq run. Our initial tests determined this to be too low for yielding sufficient material for downstream sequencing. Considering that the gene expression in our dataset varies in a similar order of magnitude as in typical scRNAseq datasets, we suggest that the amplification bias is not a dominant factor in the data. The remaining steps of the protocol were followed as in the original. More detailed methods can be found in the research protocol available at https://doi.org/10.21203/rs.3.pex-962/v1.

#### Single cell RNA-seq data analysis

Raw sequencing data (Illumina sequencer’s base call files (BCLs) was converted to Fastq files using the Illumina bcl2fastq program. The reads were trimmed to remove polyA tail and G overhang, and the 5 base UMI was extracted. The genome sequence was indexed and the single reads were aligned to the Sus scrofa reference genome sequence version Sus_scrofa.Sscrofa11.1.fasta available in the Ensembl database (RRID:SCR_002344), using STAR software (RRID:SCR_015899) STAR-2.7.2a(Dobin et al., 2013). A modified version of Sus_scrofa.Sscrofa11.1.95.gtf was used as a reference transcriptome. Feature count algorithm (featureCounts, RRID:SCR_012919), Subread R package(Liao et al., 2019) was used to count the reads to genomic features - genes and exons. These samples had an average sequencing depth of 2,591,998 reads/sample, with an average UMI count of 415,687/sample resulting in a median of 10,410 detectable genes/sample. A digital gene expression matrix was created from the gene counts. Multiple batch correction algorithm ComBat-seq(Zhang et al., 2020) was used to account for technical variability arising from batch effect. Out of 142 samples, 52 Samples with non-zero gene counts <6,000 were considered as outliers. Additionally, 10,800 genes that are present in very low quantities (<30 non-zero gene counts) were filtered out. A regularized log transformation was carried out using DESeq2(Love et al., 2014). We normalized the filtered data using a quantile regression method SCnorm(Bacher et al., 2017). Our final matrix consisted of 90 samples and 15,000 genes.

#### Extraction of significant genes from GTEx

We generated a list of neuronal genes enriched in brain and neuronal tissues using data from the Genotype Tissue Expression (GTEx) project. We downloaded the median gene expression values for all tissue types from GTEx Analysis V8 (dbGaP Accession phs000424.v8.p2). Pavlidis Template Matching was used to find genes that were specifically enriched in brain and neuronal tissues (template-maximum for neuronal tissues, minimum for non-neuronal tissues) with p value <0.01.

#### Identification of highly variable genes using PCA

Principal component analysis (PCA) was performed on 90 neuronal RNAseq samples showing expression of 15,000 genes in the RAGP. 100 genes were taken from each of the first 50 PCs, the fifty genes most positively and most negatively contributing to each PC based on PC loadings, resulting in 1,814 genes that show the most variability across the RAGP.

#### High-throughput real-time PCR

Single RAGP neurons in lysis buffer (Cells Direct Lysis Buffer, Invitrogen) were directly processed for reverse transcriptase reaction using SuperScript VILOMaster Mix (Thermo Fisher Scientific, Waltham, MA), followed by real-time PCR for targeted amplification and detection using the Evagreen intercalated dye-based approach to detect the PCR-amplified product. Intron-spanning PCR primers were designed for every assay using Primer3(Untergasser et al., 2012) and BLAST(Ye et al., 2012). Genes were selected from across a wide array of neuronal functions, signal transduction and cell type identification. The standard BioMark protocol was used to process cDNA samples for 22 cycles of specific target amplification of 283 genes using TaqMan PreAmp Master Mix as per the manufacturer’s protocol (Applied Biosystems, Foster City, CA, USA). Real-time PCR reactions were performed using 96.96 BioMark Dynamic Arrays (Fluidigm, South San Francisco, CA, USA) enabling quantitative measurement of multiple mRNAs and samples under identical reaction conditions. Each run consisted of 30 amplification cycles (15 s at 95°C, 5 s at 70°C, 60 s at 60°C). Ct values were calculated by the Real-Time PCRAnalysis Software (Fluidigm). Twenty one 96 × 96 BioMark Arrays were used to measure gene expression across all the (422 samples before QC) single-cell samples from 4 RAGP. The same serial dilution sample set was included in each chip to verify reproducibility and test for technical variability. This 6-point dilution series also serves to detect any over-amplification that may lead to a bias in the data. Samples from each animal were run across three chips to obtain data on 283 genes per sample. Each set of chip runs for a given animal contained overlapping assays that served as technical replicates to evaluate chip-to-chip variability. A chip-to-chip comparison of the serial dilution samples and neuronal/assay technical replicates demonstrates the high reproducibility with minimal technical variability of our data (Figure S10). More detailed methods can be found in the research protocol available at https://doi.org/10.21203/rs.3.pex-919/v1.

#### HT-qPCR data analysis

Individual qRT-PCR results were examined to determine the quality of the qRT-PCR based on melt-curve analysis. Following this initial quality control, samples with >30% failed reactions and genes with >20% failed reactions were excluded from present analysis. A further 10 samples were determined to be outliers due overall gene expression distributions and were removed from the present analysis. Upon filtering based on these criteria, a total of 405 single-cell samples (152 non SAN-projecting neurons, 169 SAN-projecting neurons, and 84 neurons without reliable connectivity information) and 241 different gene assays were carried forward in the present analysis, with 211 genes showing >60% detectable expression across all RAGPs, which were used for the majority of the analysis. Raw Ct values for individual samples were normalized against a median expression level of a subset of 140 robustly expressed genes (genes with greater than 60% working reactions) across all animals to obtain -DCt values.The vector of median sample expression value was chosen over potential reference genes based on comparison of stable expression across all samples against known housekeeping genes using the ‘selectHKs’ function in the NormqPCR package in R (RRID:SCR_003388). The following equation was used to calculate -DCt values for each gene:$-DCt_{gene}\;=\;\left(median\;sample\;expression\right)\;-\;Ct_{gene}$.

The -DCt data were then rescaled using the median across all samples within a gene using the following equation:

$$-DDCt_{gene}=-\left(DCt_{sample}-\;DCt_{across-sample-median}\right).$$

The raw and normalized dataset is available online as a Gene Expression Omnibus (RRID:SCR_005012) dataset (GEO reference ID: GSE149212) and on sparc.science (RRID:SCR_017041) portal at https://doi.org/10.26275/5jki-b4er.

#### Determination of abundant/detectable expression

We considered a sample to have detectable expression of any given gene if any signal was detected in the HT-qPCR assay. A sample was considered to have abundant expression if its expression of that gene was less than 15 Ct. One exception is the expression level of Chat. The levels of Chat in the second female RAGP (Pig 1729) had a median expression of 7 Ct higher than that of the other 3 RAGP. Despite the reduced expression of Chat in raw Ct, it is important to note that the range of expression remains the same despite the decrease in abundance. Due to this consistency in range and pattern, we considered samples from Pig 1729 to have abundant expression if it had a Ct less than 22, consistent with the 7 Ct difference between the medians of expression.

#### Clustering and template matching to identify transcriptional states

Single neurons were separated into different subpopulations based on their molecular profiles using Pavlidis Template Matching. Briefly, template matching estimates the correlation between a rescaled expression profile used as the template and a test expression profile. The canonical subpopulations were identified in SAN-projecting samples from one female and one male RAGP using hierarchical clustering (Pearson correlation, complete linkage) yielding seven sample clusters. The average silhouette width for all clusters (estimated using the cluster package in R) was 0.217, significantly higher than 1,000 randomized trials. Gene medians of these clusters were used as templates to classify the non SAN-projecting cells into one of the canonical subpopulations. We used an R-based cutoff for our template match analysis (threshold R = 0.45). Single neurons that did not pass the R value threshold for any of the canonical templates were sequestered into a separate cluster, yielding 8 clusters total. Samples from another male (with cells annotated for projection) and female (without cells annotated for projection) were sorted into the original states based on correlation to the template profile (Figures 4A andS3). Finally, based on visual examination, three states were combined to form state A based on their similar expression profiles, giving 6 total transcriptional states. An enrichment assessed by a one-way ANOVA and post hoc Tukey Honest Significant Difference was used to examine genes that were significantly enriched in one or more states. A gene was considered to be enriched in a state if p value <0.01 for at least two other states (Figures 4D–4F).

#### Plots for showing abundance and range of gene expression in Ct space

In order to accurately display the range of the normalized data while also keeping in mind the overall abundance of each gene throughout the 4 RAGP, the -ΔΔCt data was visualized in Ct space. To do this, we first confirmed that the median Ct of each gene was relatively consistent across all 4 RAGP. The normalized expression for each animal was multiplied by -1 and then offset by the median gene expression across all 4 RAGP. This resulted in a plot that displayed expression level in Ct space (where the lower the Ct the higher the expression), showing the overall abundance of the gene in question across 4 RAGP while maintaining the normalized range of expression (across all RAGP for some figures, and comparing the ranges of each RAGP for other figures).

#### Paracrine network analysis

Different paracrine signaling states of single neurons in the RAGP were created from the combination of identifiers that describe whether a neuron is more likely to transmit a neuropeptide signal, be activated by that signal, or both. Each neuron was identified as peptide+ or peptide-based on whether or not the neuron in question expressed the given peptide above or below the median expression value for that gene. Likewise, each neuron was identified as receptor+ if any one of the corresponding receptors showed expression above the median value for either receptor. Applying these identifiers to each peptide-receptor set resulted in 8 possible unique identifiers for each set, which when combined resulted in 64 possible unique identifiers. After applying these identifiers to the 405 sampled neurons, at least one neuron was assigned to 61 out of the 64 possible identifiers, indicating a large and widespread network of neuropeptide signaling, even when examining only 3 neuropeptides and their cognate receptors (Data S1). Of those 61 states, 12 groups have a frequency of more than 10 sampled neurons, representing a total of 224 out of the 405 single neurons, about 55%. A network representation of these 12 identified groups further highlights the interconnected nature of signaling between neurons.

#### Confocal staining and visualization

Porcine RAGP fat pads were fixed in cold 4% paraformaldehyde in PBS for 24 h, cryoprotected in cold 20% sucrose in PBS, and sectioned at 30 mm thickness using a Leica CM3050S cryostat (Leica Microsystems Inc., Bannockburn, IL, USA). Sections were collected on charged slides and immunostained at room temperature using standard methods of fluorescence immunohistochemistry, as described previously(Hoard et al., 2008; Hoover et al., 2009; Kaestner et al., 2019). Antibodies to vesicular acetylcholine transporter (VAChT; Synaptic Systems, Cat. No. 139103, RRID: AB_887864,1:500 dilution) and tyrosine hydroxylase (TH; Millipore, Cat. No. AB1542, RRID:AB_90755, 1:500 dilution) were used to label cholinergic and noradrenergic neurons, respectively. The VAChT antibody was generated in rabbit using aa 475-530 from rat VAChT as the immunogen, and it has been validated by experiments with knockout and knockdown mice as well as by immunohistochemistry and Western blotting. The TH antibody was generated in sheep using native TH from rat pheochromocytoma, and it has been validated by immunohistochemistry and Western blotting. Sections were washed and blocked before incubation overnight with both primary antibodies. This was followed by washing and blocking again before addition of species-specific donkey secondary antibodies conjugated to Alexa Fluor 488 for VAChT and biotin-SP for TH (Jackson ImmunoResearch Laboratories). After incubation for 2 h with secondary antibodies and washing with PBS, sections were incubated with streptavidin conjugated Cy3 (Jackson ImmunoResearch Laboratories) to amplify the TH signal. Sections were then washed with PBS, and cover glasses were applied with Citifluor (Ted Pella, Inc.) or SlowFade Gold antifade reagent (Life Technologies Corporation, Eugene, OR, USA). Cover glasses were sealed with clear nail polish. Specific staining did not occur in negative control sections processed without the addition of the primary antibodies.The localization of VAChT and TH was evaluated by confocal microscopy with a Leica TCS SP8 Confocal Microscope (Leica Microsystems Inc.). Confocal images were collected at a resolution of 1024X1024 by sequential scans using 488 and 552 laser lines. Stacks of optical sections, with the section number optimized by the software, were collected for regions of interest using 4-line averages for each scan. Stacks spanned the entire tissue thicknesses. Figures were created using maximum intensity projection images for individual channels and merged images. These images compress the stack of optical images into a two-dimensional view by showing only the highest intensity pixel across the stack for each point.

### Quantification and statistical analysis

All statistical analysis was done using the ‘R’ programming language. Pavlidis Template Matching was used to find genes that were specifically enriched in the brain and neuronal tissues from GTEx data with a p value <0.01. Pavlidis Template Matching was also used to to separate single neurons into different subpopulations and a subsequent ANOVA and post hoc Tukey Honest Significant Difference was used to examine genes that were enriched in one or more subpopulations for p < 0.01. More information regarding statistical analysis can be found within each figure legend and the accompanying sections within the method details.

## Supplemental information

Document S1. Figures S1–S10

Data S1. Supplemental Cytoscape network file, related to Figure 6 Cytoscape file containing all the network information related to Figure 6 and Video S4. This Cytoscape session file includes annotated networks corresponding to the individual neuropeptides as well as the combined neuropeptidergic network mentioned in the text and the simplified network shown in Figure 6.
