# Supplementary material for: A single cell transcriptomics map of paracrine networks in the intrinsic cardiac nervous system
Source: iScience. 2021 Jul 19;24(7):102713. doi: 10.1016/j.isci.2021.102713 (PMC8324809; doi:10.1016/j.isci.2021.102713)
Supplement: Document S1. Figures S1–S10 [file mmc1.pdf]

iScience, Volume *24*

Supplemental information

A single cell transcriptomics map of paracrine networks in the intrinsic cardiac nervous system

Alison Moss, Shaina Robbins, Sirisha Achanta, Lakshmi Kuttippurathu, Scott Turick, Sean Nieves, Peter Hanna, Elizabeth H. Smith, Donald B. Hoover, Jin Chen, Zixi (Jack) Cheng, Jeffrey L. Ardell, Kalyanam Shivkumar, James S. Schwaber, and Rajanikanth Vadigepalli

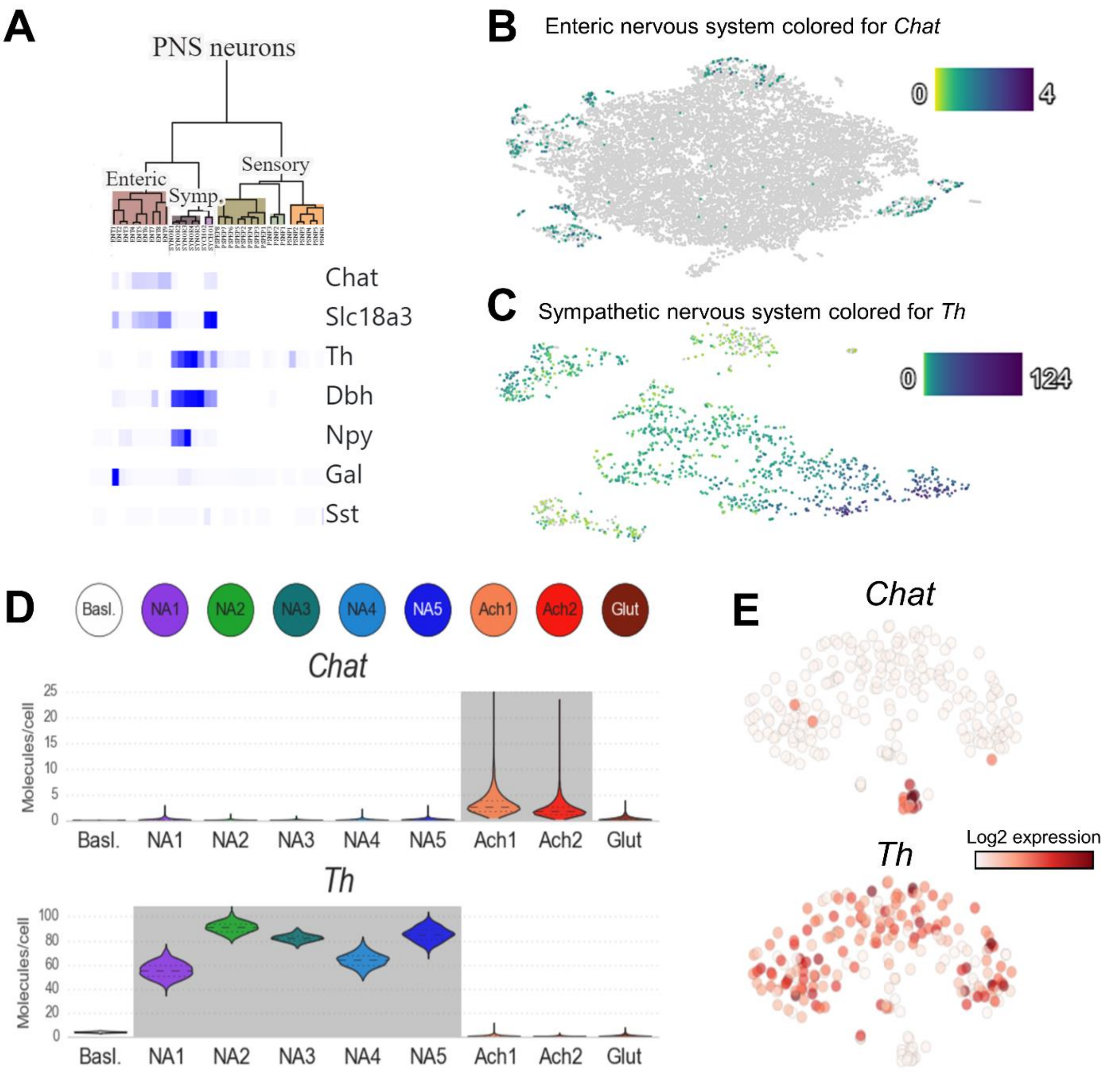

Figure S1: Analysis of the co-expression patterns of *Chat* and *Th* in the mouse peripheral nervous system (Zeisel et al. 2018, Furlan et al. 2016), related to Figure 2. (A) Dendrogram describing identified cell types in the PNS and indicating the relative abundance of the indicated genes across those cell types(Zeisel et al., 2018) (http://mousebrain.org/genesearch.html). **(B,C)** tSNE for the enteric nervous system (Mousebrain.org.level1/L1_Enteric.loom) (B) and the sympathetic ganglia (Mousebrain.org.level1/L1_Sympathetic.loom) (C) colored for expression of *Chat* and *Th*, respectively. Of note, the enteric nervous system does not present as distinct cell types within the context of the tSNE, suggesting gradients of expression across the neuronal phenotype landscape where expression of *Chat* is concentrated in areas around the outside. While the sympathetic nervous system presents as more clustered, there still exists a gradient of *Th* expression across the neuronal phenotype landscape(Zeisel et al., 2018) **(D)** Violin plots showing expression of *Chat* (top) and *Th* (bottom) in identified noradrenergic (NA1-5) and cholinergic (Ach1-2) cell types in sympathetic ganglia(Furlan et al., 2016). **(E)** tSNE of single cells in sympathetic ganglia colored for expression of *Chat* (top) and *Th* (bottom)(Furlan et al., 2016) (http://linnarssonlab.org/sympathetic/).

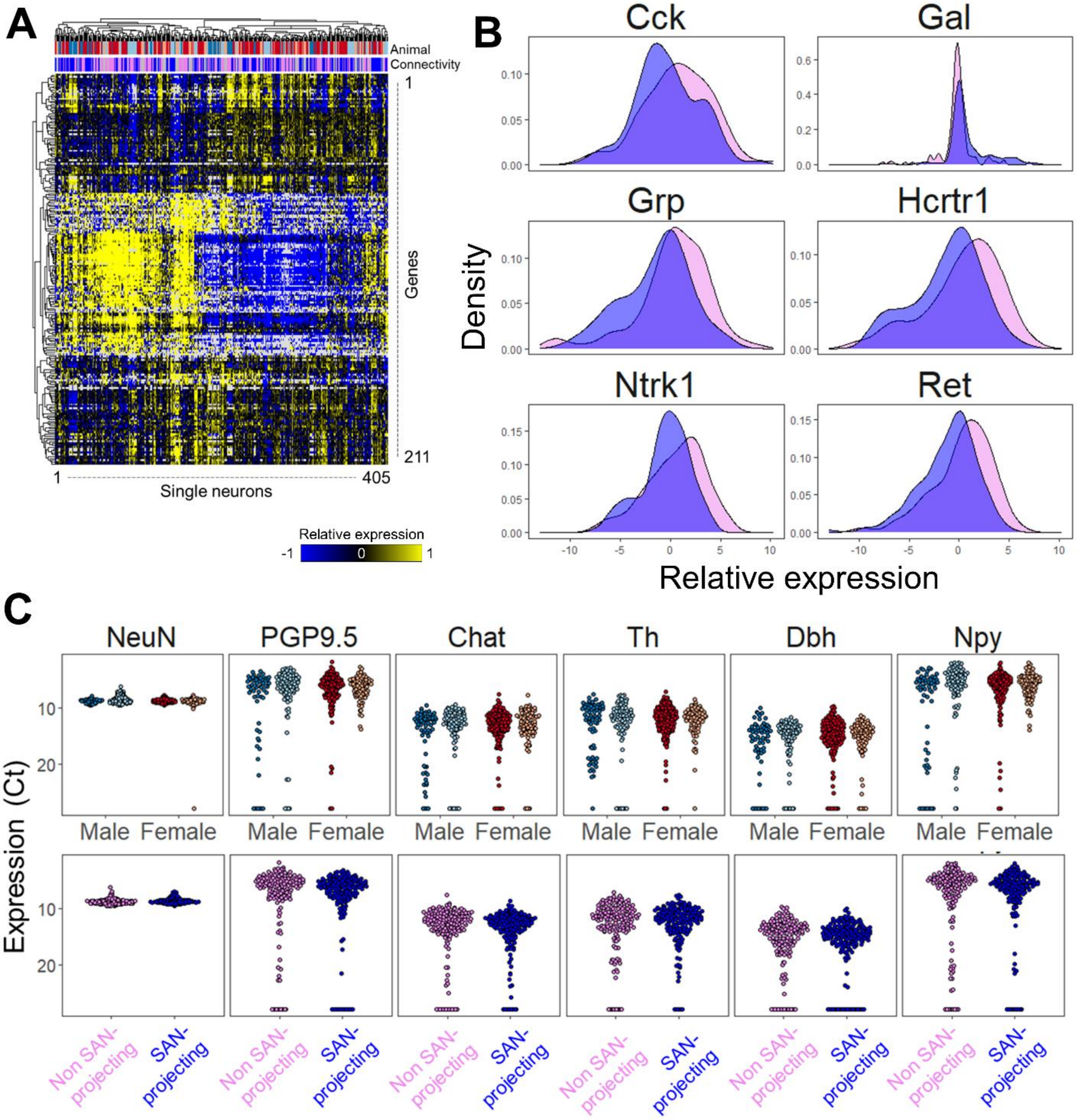

Figure S2: Expression of 405 single neuron samples across RAGP and between SAN-projecting and non SAN-projecting neurons, related to Figure 4. (A) Expression of 211 genes assayed through HT-qPCR that have >60% detectable expression across 405 single neurons (n=4 animals). **(B)** Density distribution of SAN-projecting and non SAN-projecting single neurons for 6 genes that show statistical differences between projecting and non-projecting neurons (K-S statistic, FDR-adjusted $p < 0.01$, fold change > 2). **(C)** Expression distribution showing both the range and abundance of select neuronal markers across RAGP (top) and between SAN-projecting and non SAN-projecting neurons (bottom).

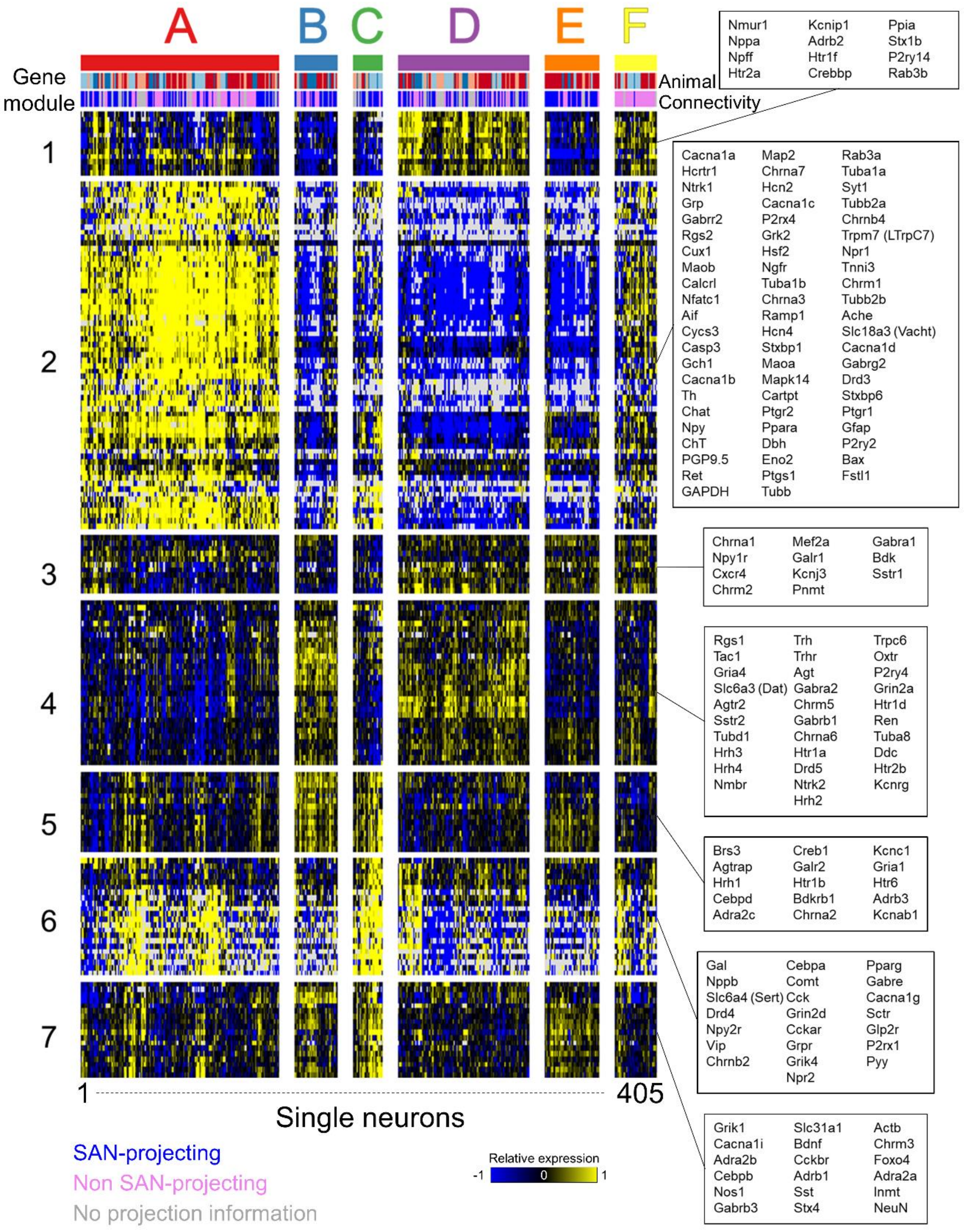

Figure S3: Landscape of neuronal transcriptional states across 4 RAGP, related to Figure 4. Expression of 174 genes each assayed through HT-qPCR in 405 single neurons (n=4 animals). Accompanies Figure 4A which shows expression of 321 single neurons (n=3 animals). A fourth pig with no projection information was further incorporated into the identified transcriptional states. Genes names in each box are organized in the same order of the genes in the heatmap, to be read down each column.

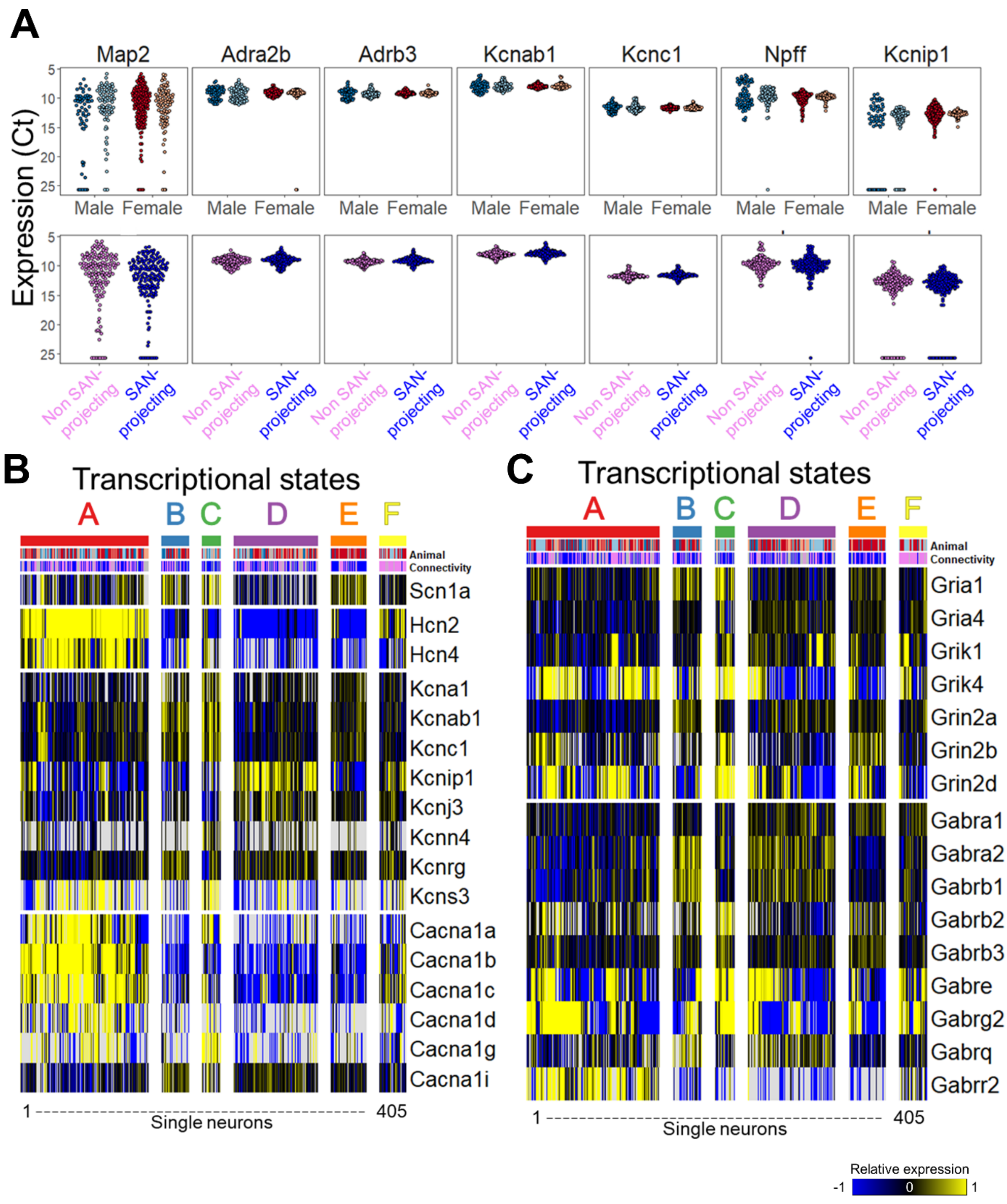

Figure S4: Expression patterns of key genes that differentiate transcriptional states and ion channels and transporters across single neurons in RAGP, related to Figure 4. (A) Expression distribution showing both the range and abundance for selected genes found to be enriched in a particular transcriptional state (Figure 4D-F). Expression is shown across RAGP (top) and between SAN-projecting and non SAN-projecting neurons (bottom). **(B,C)** Gene expression of various sodium, potassium, and calcium channels (B) and various glutamatergic and GABAergic receptors (C) across the identified transcriptional states.

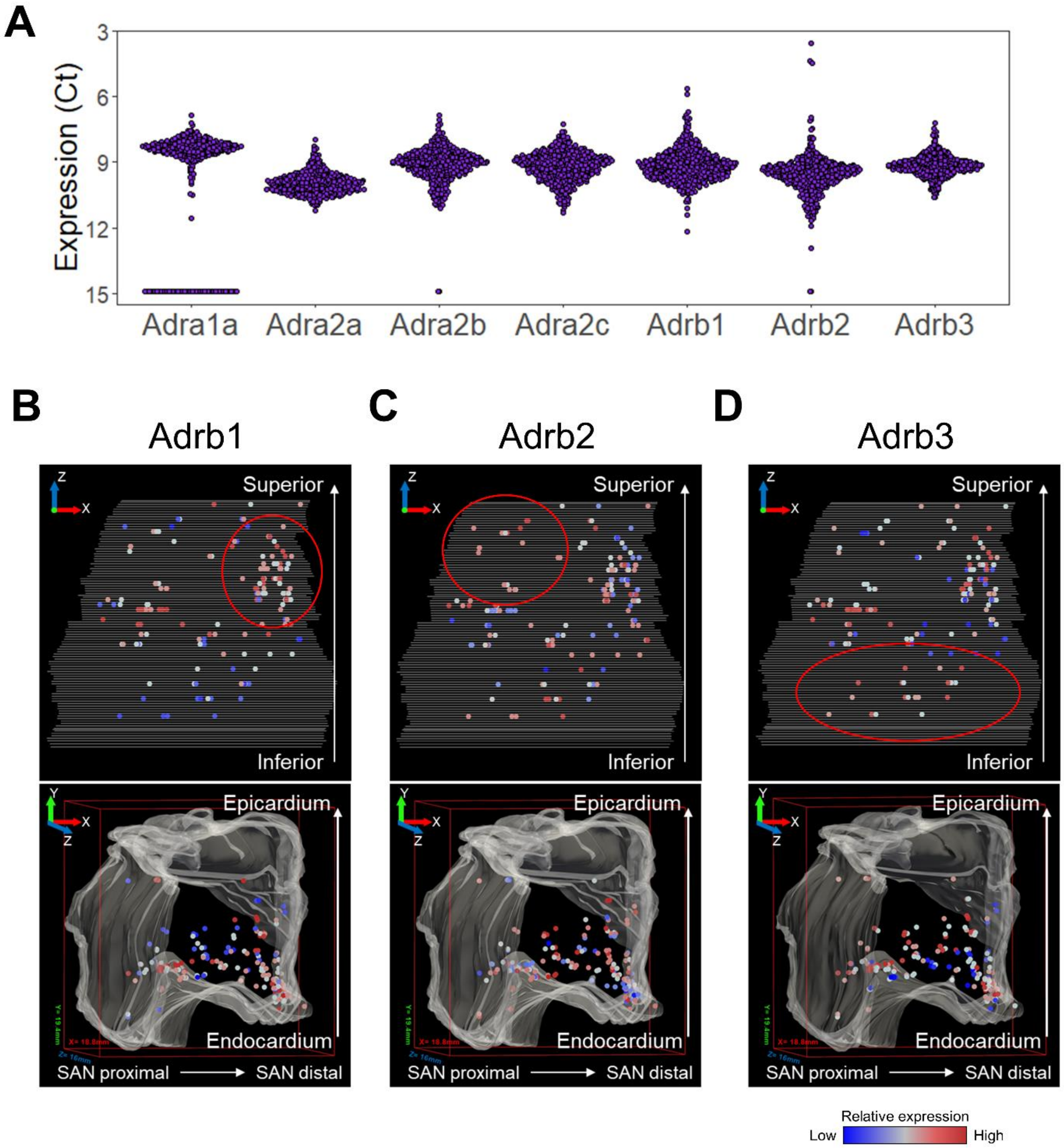

Figure S5: Expression of adrenergic receptors in the RAGP, related to Figure 4. (A) Beeswarm plot showing the abundance and the range of expression of adrenergic receptors across 405 single RAGP neurons (n=4 animals). **(B-D)** Visualization of *Adrb1* (B), *Adrb2* (C), and *Adrb3* (D) gene expression within the 3D anatomical framework for a representative RAGP. The red circles demarcate regions of high expression. The bounding box on the lower panel shows 18.8 mm, 19.4 mm, and 16 mm on the X, Y, and Z axis, respectively.

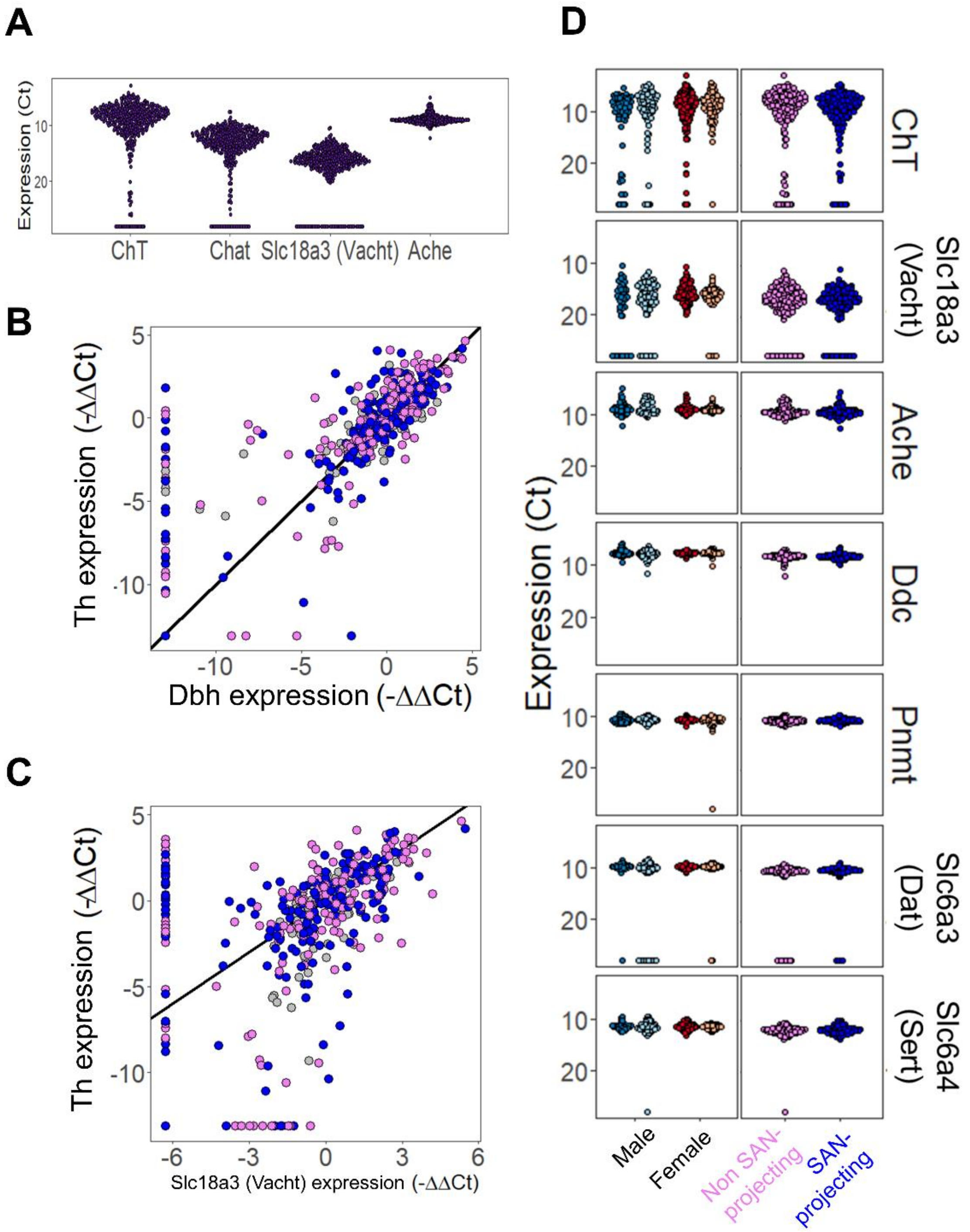

Figure S6: Expression of cholinergic and catecholaminergic markers, related to Figure 5. (A) Beeswarm plot showing the abundance and the range of expression of key genes involved in acetylcholine biosynthesis and transport across 405 single RAGP neurons (n=4 animals). **(B-C)** Co-expression of *Th* with *Dbh* (B) *and Slc18a3 (Vacht)* (C) across 405 single RAGP neurons (n=4 animals). Solid line represents a diagonal of equal expression. **(D)** Expression distribution showing both the range and abundance for genes involved in biosynthesis and transport of catecholamines and acetylcholine. Expression is shown across RAGP (top) and between SAN-projecting and non SAN-projecting neurons (bottom). Remaining genes in these pathways can be found in Figure S2C.

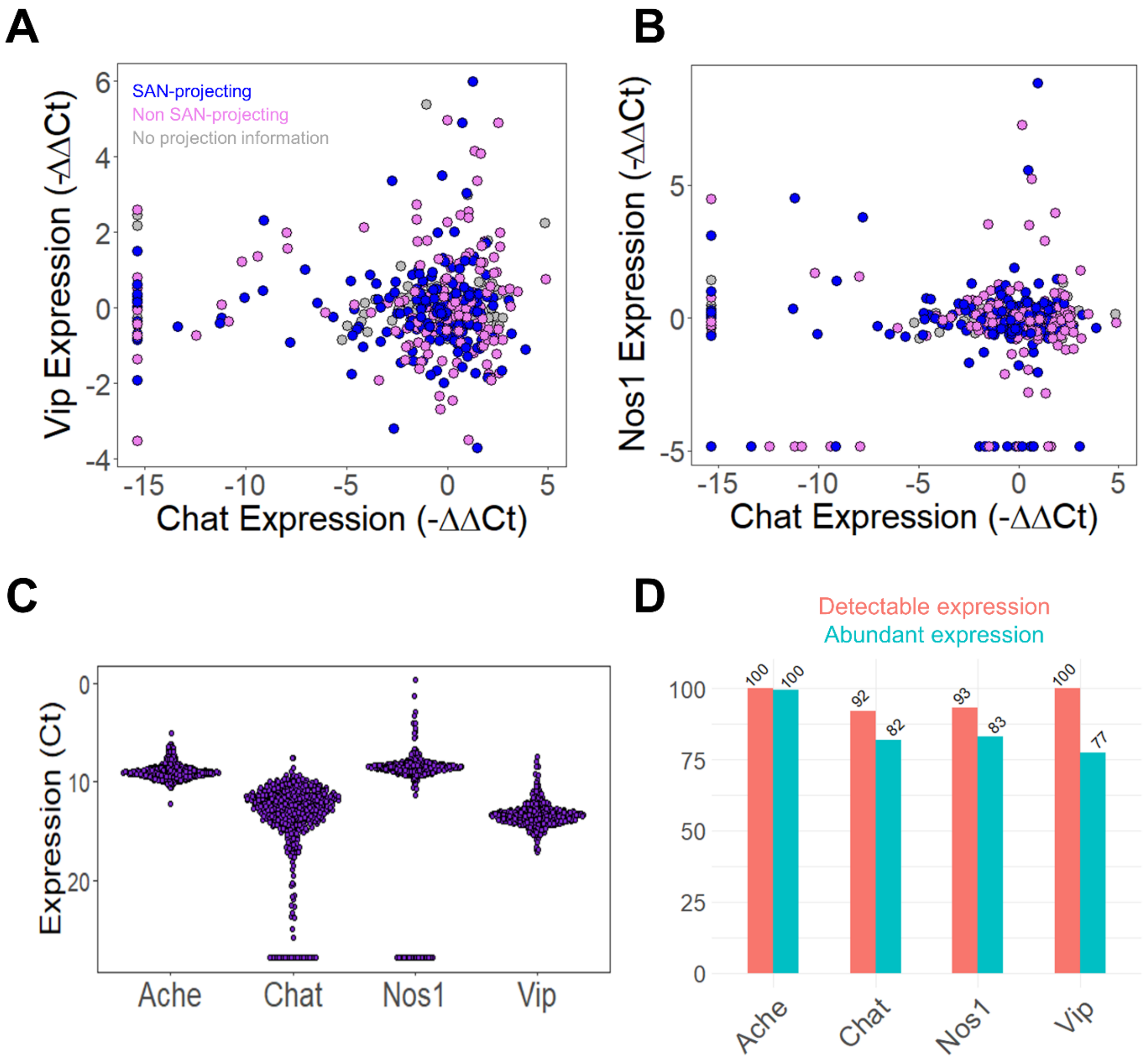

Figure S7: Expression of Nos1 and Vip, related to Figure 5. (A,B) Co-expression of Chat with Vip (A) and Nos1 (B) across 405 single RAGP neurons (n=4 animals). **(C)** Beeswarm plot showing the abundance and the range of Ache, Chat, Nos1, and Vip across 405 single RAGP neurons (n=4 animals). **(D)** Proportion of samples that showed detectable and abundant expression of select genes (n= 4 animals).

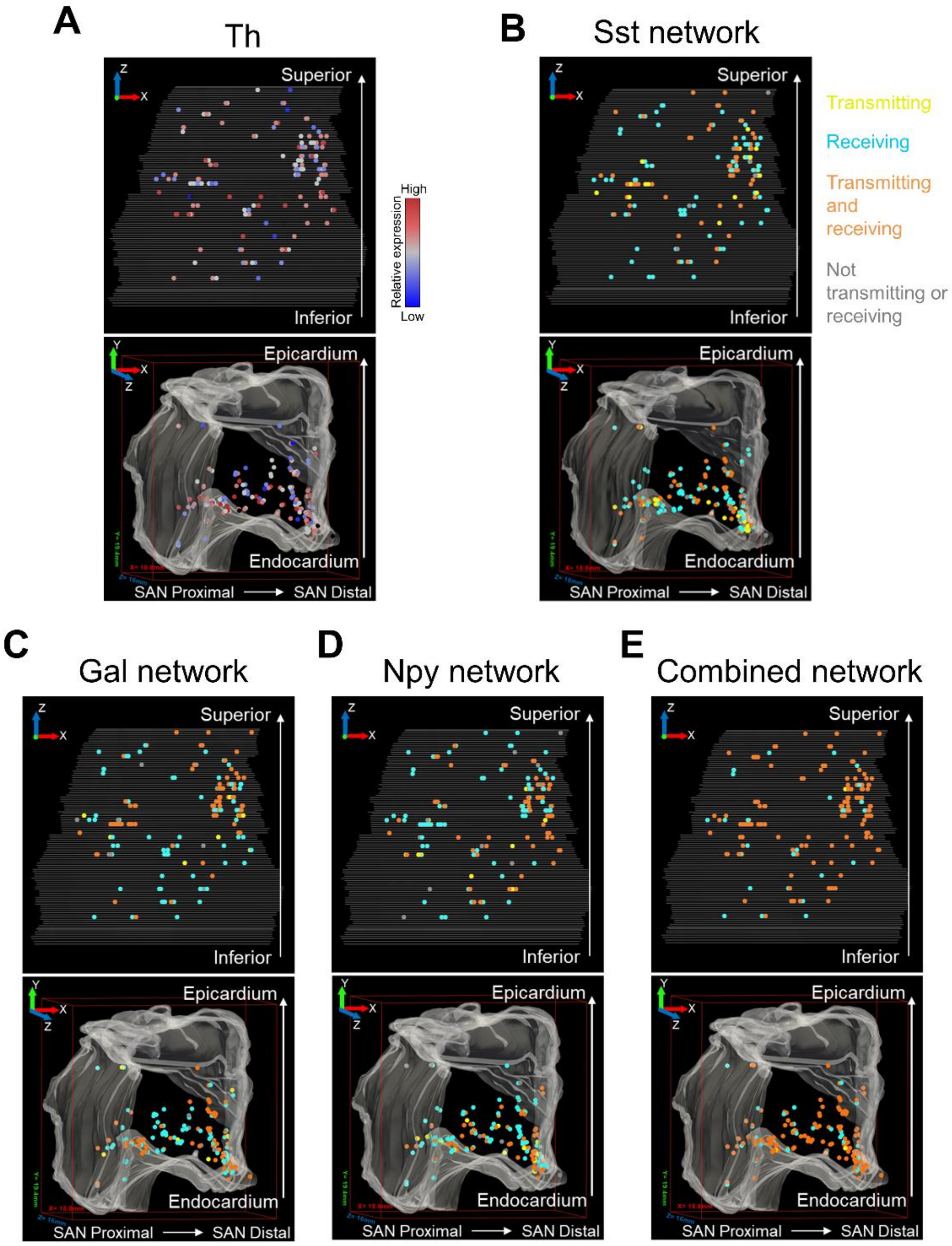

Figure S8: 3D visualization of a representative RAGP, related to Figure 5,6. (A) Visualization of *Th* gene expression within the 3D anatomical framework for a representative RAGP. **(B-E)** Visualization of local paracrine networks for *Sst* (B), *Gal* (C), and *Npy* (D), as well as the combined network (E), within the 3D anatomical framework for a representative RAGP. The bounding box on the lower panel shows 18.8 mm, 19.4 mm, and 16 mm on the X, Y, and Z axis, respectively.

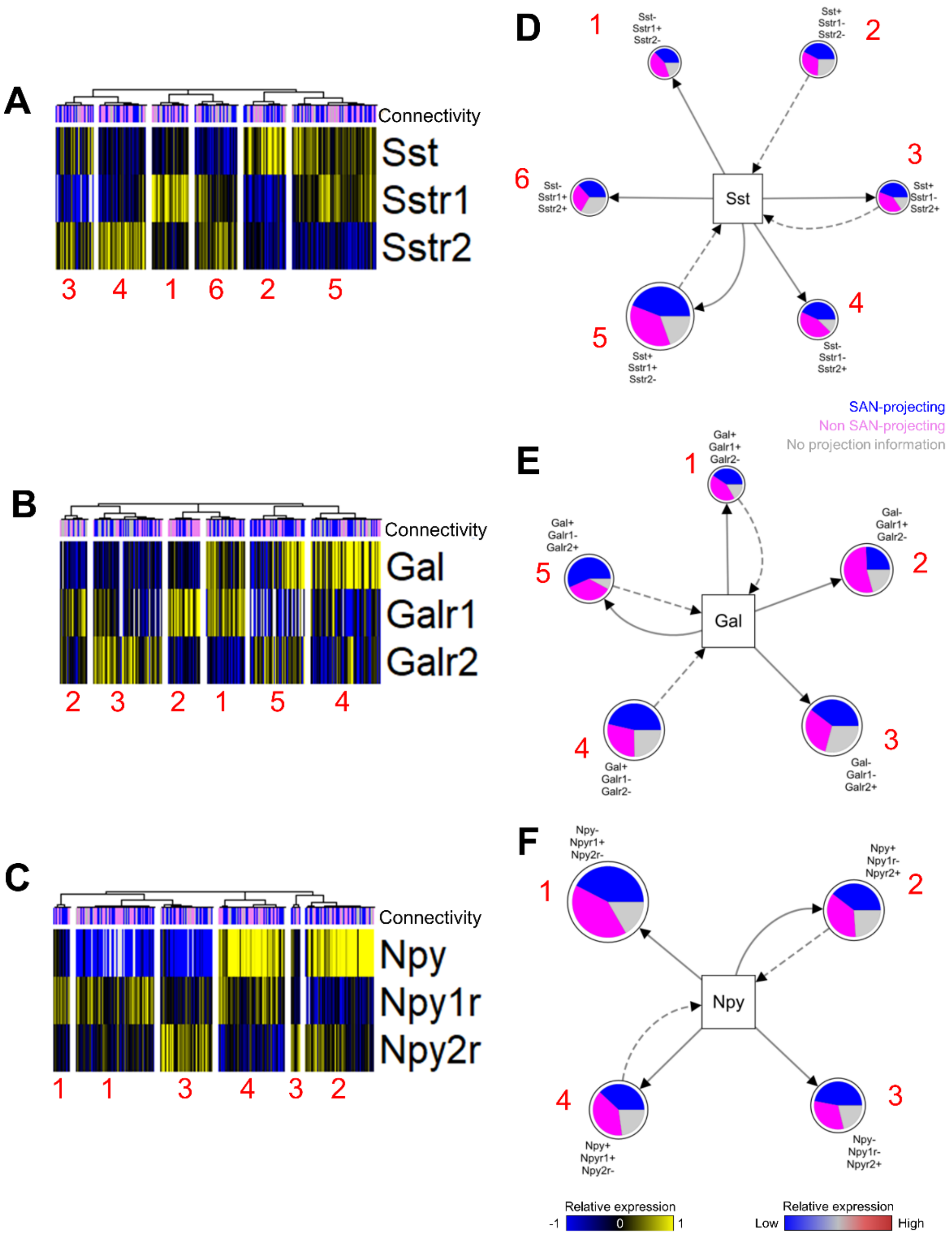

Figure S9: Neuropeptidergic interaction networks in the pig RAGP, related to Figure 6. (A-C) Expression patterns of somatostatin (A), galanin (C), and neuropeptide Y (E) and their cognate receptors across 405 single RAGP neurons (n=4 animals) that are numbered corresponding to their representative node in the networks for (D-F). **(D-F)** Network representations of the heatmaps from (A,C,E) where each cluster is represented as a specific node depending on whether or not it is transmitting the peptide signal or receiving it through one of the cognate receptors. The pie chart within each circular node indicates the proportion of the neurons within that subtype that are identified as projecting to the SAN region. The size of the node is proportional to the number of single neurons belonging to each subtype.

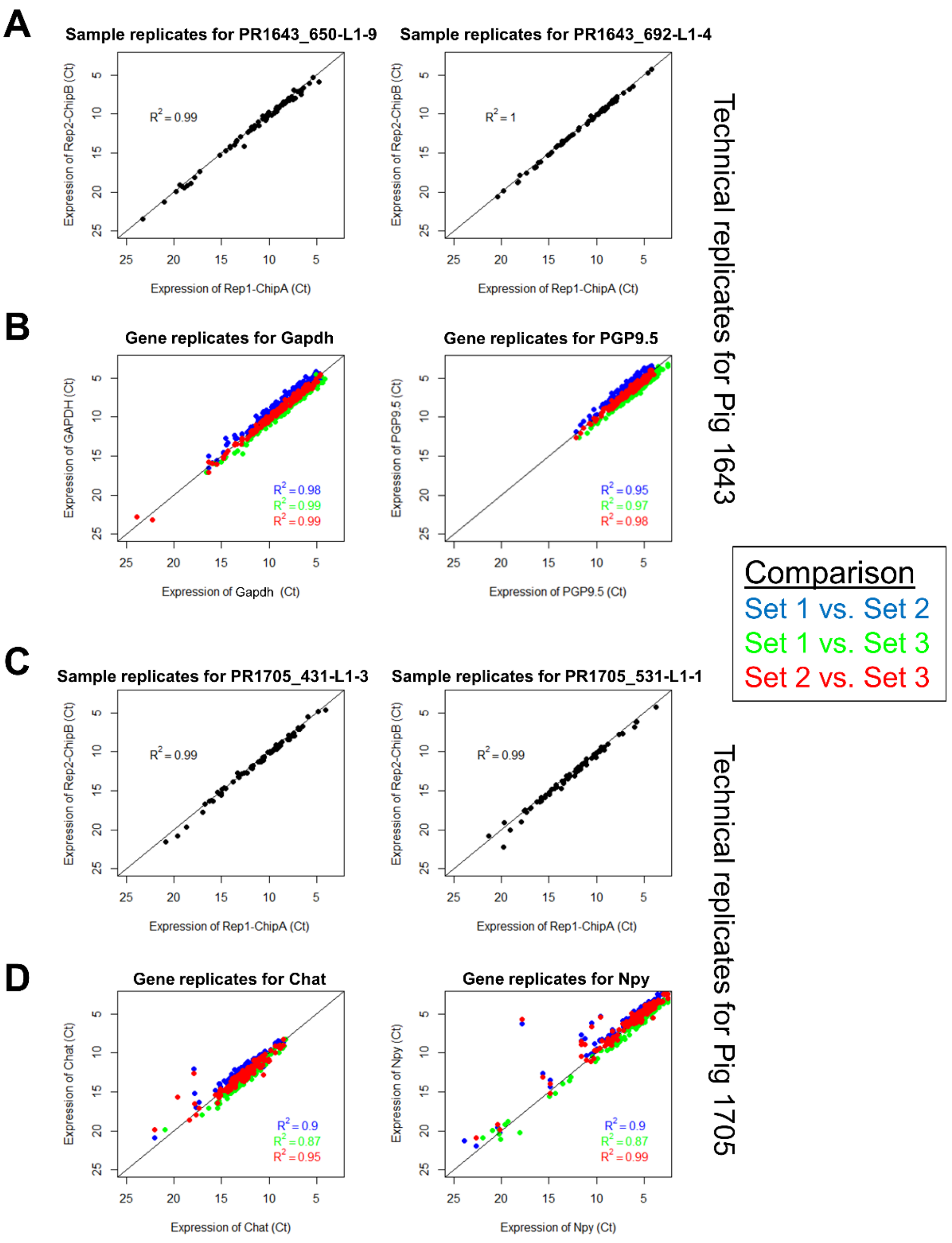

Figure S10: Technical reproducibility of HT-qPCR, related to STAR Methods, section High-throughput real-time PCR. (A-D) Technical Replicates of Samples and Genes in Pig 1643 (A-B) and Pig 1705 (C-D). To check for technical reproducibility within the HT-qPCR data, select samples and genes were run on multiple BioMark Chips. Samples had two replicates (A,C) and genes had three replicates (B,D). To robustly assess the three gene replicates, we compared each of the three sets to each other, shown in three different colors, where blue represents the comparison between sets 1 and 2, green represents the comparison between sets 1 and 3, and red represents the comparison between sets 2 and 3.
